# Comparison of dot chromosome sequences from *D. melanogaster *and *D. virilis *reveals an enrichment of DNA transposon sequences in heterochromatic domains

**DOI:** 10.1186/gb-2006-7-2-r15

**Published:** 2006-02-20

**Authors:** Elizabeth E Slawson, Christopher D Shaffer, Colin D Malone, Wilson Leung, Elmer Kellmann, Rachel B Shevchek, Carolyn A Craig, Seth M Bloom, James Bogenpohl, James Dee, Emiko TA Morimoto, Jenny Myoung, Andrew S Nett, Fatih Ozsolak, Mindy E Tittiger, Andrea Zeug, Mary-Lou Pardue, Jeremy Buhler, Elaine R Mardis, Sarah CR Elgin

**Affiliations:** 1 Biology Department, Washington University, St Louis, MO 63130, USA; 2 Member, Bio 4342 class, Washington University, St Louis, MO 63130, USA; 3 Department of Biology, Massachusetts Institute of Technology, Cambridge, MA 02139, USA; 4 Computer Science and Engineering, Washington University, St Louis, MO 63130, USA; 5 Genome Sequencing Center and Department of Genetics, Washington University, St Louis, MO 63108, USA

## Abstract

**Background:**

Chromosome four of *Drosophila melanogaster*, known as the dot chromosome, is largely heterochromatic, as shown by immunofluorescent staining with antibodies to heterochromatin protein 1 (HP1) and histone H3K9me. In contrast, the absence of HP1 and H3K9me from the dot chromosome in *D. virilis *suggests that this region is euchromatic. *D. virilis *diverged from *D. melanogaster *40 to 60 million years ago.

**Results:**

Here we describe finished sequencing and analysis of 11 fosmids hybridizing to the dot chromosome of *D. virilis *(372,650 base-pairs) and seven fosmids from major euchromatic chromosome arms (273,110 base-pairs). Most genes from the dot chromosome of *D. melanogaster *remain on the dot chromosome in *D. virilis*, but many inversions have occurred. The dot chromosomes of both species are similar to the major chromosome arms in gene density and coding density, but the dot chromosome genes of both species have larger introns. The *D. virilis *dot chromosome fosmids have a high repeat density (22.8%), similar to homologous regions of *D. melanogaster *(26.5%). There are, however, major differences in the representation of repetitive elements. Remnants of DNA transposons make up only 6.3% of the *D. virilis *dot chromosome fosmids, but 18.4% of the homologous regions from *D. melanogaster*; *DINE-1 *and *1360 *elements are particularly enriched in *D. melanogaster*. Euchromatic domains on the major chromosomes in both species have very few DNA transposons (less than 0.4 %).

**Conclusion:**

Combining these results with recent findings about RNAi, we suggest that specific repetitive elements, as well as density, play a role in determining higher-order chromatin packaging.

## Abstract 2

Sequencing and analysis of fosmid hybridization to the dot chromosomes of *Drosophila virilis *and *D. melanogaster *suggest that repetitive elements and density are important in determining higher-order chromatin packaging.

## Background

FDNA in the eukaryotic interphase nucleus can broadly be distinguished as packaged into two different forms of chromatin, heterochromatin and euchromatin [1]. Classically, heterochromatin has been described as the fraction that remains highly condensed in interphase, has high affinity for DNA-specific dyes, and is commonly seen around the periphery of the nucleus [2]. Heterochromatic regions of the genome have very low rates of meiotic recombination and generally replicate late in S phase. These regions are rich in repetitive sequences, including remnants of transposable elements and retroviruses, as well as simple repeats (satellite DNA). Heterochromatin tends to be gene poor, and those genes found in heterochromatin tend to be larger (longer transcripts) than genes found in euchromatin [3]. Introns of heterochromatic genes have a much higher density of transposable elements than introns of euchromatic genes, accounting for this shift [4]. The less densely packaged euchromatin contains most of the actively transcribed genes. In contrast to this general picture of repeat distribution, Pardue *et al*. [5] have found by *in situ *hybridization that the frequency of (dC-dA)·(dG-dT) dinucleotide repeats is higher in euchromatin than in heterochromatin.

Several biochemical marks have been identified that distinguish heterochromatin from euchromatin, including a distinctive pattern of histone modification and the association of particular chromosomal proteins [6]. High concentrations of heterochromatin protein 1 (HP1) are found primarily in pericentric heterochromatin and associated with telomeres in organisms from the yeast *Schizosaccharomyces pombe *to mammals [7,8]. Histones in euchromatic domains are typically hyperacetylated, particularly the amino-terminal tails of H3 and H4. In contrast, methylation of histone H3 at lysine 9 (producing H3K9me) is a consistent mark of heterochromatin [9]. HP1 binds to H3K9me through its chromo domain and to SU(VAR)3-9, a methyltransferase that specifically modifies histone H3 at K9, through its chromo shadow domain [9,10]. These interactions are thought to contribute to heterochromatin maintenance and spreading [1]. The functional significance of this chromatin packaging is demonstrated by the observation that loss-of-function mutations in the gene for HP1, including one that disrupts binding of HP1 to H3K9me, result in a loss of silencing of reporter genes placed in or near heterochromatin (suppression of position effect variegation) [11].

Chromosome four of *Drosophila melanogaster*, also known as the dot chromosome or the F element, is unique in its chromatin composition. The banded portion (amplified during polytenization) is 1.2 Mb long with 82 genes; this gene density is similar to that of the euchromatic regions of the major (euchromatic) chromosome arms [12,13]. However, the fourth chromosome also displays many characteristics of heterochromatin, including late replication [14] and a complete lack of meiotic recombination [15]. The banded region of chromosome 4 is known to have an approximately ten-fold higher density of repetitive elements (for example, remnants of retroviruses, transposable elements) in comparison with the long arms of chromosomes 2, 3, and X [16-19], but has little or no (dC-dA)·(dG-dT) dinucleotide repeats [5], again resembling heterochromatin rather than euchromatin. Immunofluorescent staining of polytene chromosomes with antibodies directed against HP1 shows an abundance of HP1 in a banded pattern on chromosome four [20]. A very similar pattern is seen with antibodies directed against H3K9me [9,21].

A transposable *P *element containing an *hsp70*-driven *white *(*w*) gene has been a useful reporter of chromatin packaging, giving a uniform red eye phenotype when inserted into the euchromatic arms but a variegating phenotype when inserted into the pericentric heterochromatin or into telomere associated sequences [22]. The variegating phenotype is associated with packaging into a nucleosome array showing more uniform spacing, accompanied by a loss of DNase hypersensitive (DH) sites [23]. Transposition events resulting in insertions on the fourth chromosome produce both variegating and solid red eye phenotypes. The data suggest that while the fourth chromosome of *D. melanogaster *is largely heterochromatic, it also includes some euchromatic domains [23].

*P *element transposition-induced deletions and duplications of small genomic regions around the genes *Hcf *and *CG2052 *on chromosome four have been shown to cause switching of eye phenotypes from red to variegating and vice versa [24]. Mapping of the breakpoints has shown that the small deletions and duplications lead to changes in the distance of the reporter from a particular DNA transposon, *1360 (*also known as *hoppel *or PROTOP_A). In the region of the fourth chromosome studied, if the inserted *P *element is within approximately 10 kilobases (kb) of a *1360 *element, the *white *reporter gene has a greater than 90% chance of exhibiting variegating expression, suggesting it is in a heterochromatic domain. If the reporter is more than 10 kb away from a *1360 *element, it has a greater than 90% chance of generating a red eye phenotype, suggesting that it is in a euchromatic domain. Therefore, Sun *et al*. [24] have suggested that proximity to the *1360 *element can influence the chromatin packaging state. Recent results from fungi and plants [25], as well as *Drosophila *[26] have shown that heterochromatin formation is dependent on the RNA interference (RNAi) system. Small double-stranded (ds)RNAs have been recovered from many of the repetitive elements in *Drosophila*, including *1360 *[27], and might target repetitive elements in the genome for silencing by initiation and spreading of heterochromatin packaging.

The small dot chromosome exists in many species of *Drosophila *[28]. It has long been recognized that phenotypes of similar mutations map to the dot chromosomes of both *D. melanogaster *and *D. virilis *[29,30]. Podemski *et al*. [31] have shown that probes for several genes from the *D. melanogaster *fourth chromosome, including *ci *and *Caps*, hybridize to the dot chromosome in *D. virilis*. *D. virilis *is a member of a *Drosophila *genus that diverged from *D. melanogaster *40 to 60 million years ago [32]. In addition to the sex chromosomes, it has four large autosomes, rather than the two of *D. melanogaster*; thus, the dot chromosome of *D. virilis *is chromosome six. The polytenized regions of both dot chromosomes are similar in size. In this study, we will refer to chromosome six of *D. virilis *and chromosome four of *D. melanogaster *as dot chromosomes. Our analysis concerns the banded 1.2 Mb region of these chromosomes, estimated to contain approximately 80 genes.

**Figure 1:**
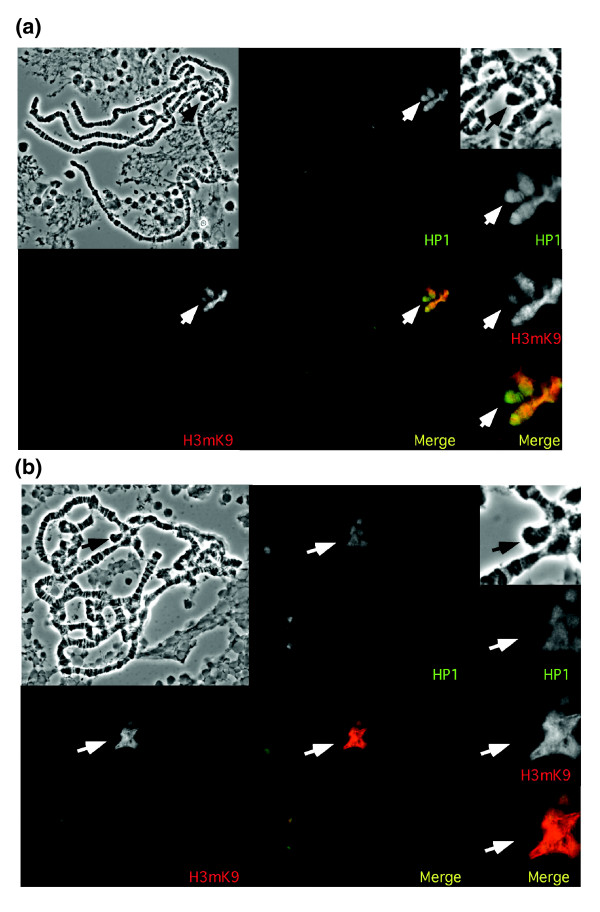
Immunofluorescent staining of the polytene chromosomes. Polytene chromosomes from **(a) ***D. melanogaster *and **(b) ***D. virilis *are shown. Top left, phase contrast; others as labeled. Panels on the right provide a close-up of the chromocenter and the dot chromosome. In the merge picture, yellow represents equal staining, red represents more H3K9me staining, and green represents more HP1 staining. The dot chromosome is indicated with an arrow. In *D. melanogaster*, antibodies for HP1 and H3K9me stain both the chromocenter and the dot chromosome, although the HP1 staining is slightly stronger than the H3K9me staining on the dot. In *D. virilis*, both antibodies stain the chromocenter but neither stains the dot chromosome.

Prior reports indicated that the dot chromosome of *D. virilis *does not share the heterochromatic characteristics of the dot chromosome of *D. melanogaster*, despite the fact that it maintains a similar proximity to the heterochromatic chromocenter, as seen in polytene nuclei. *In situ *hybridizations performed by Lowenhaupt *et al*. [33] demonstrated that the (dC-dA)·(dG-dT) dinucleotide repeat frequency of the *D. virilis *dot chromosome is similar to that in its euchromatic arms. In contrast to the observations using *D. melanogaster*, recombination is observed on the *D. virilis *dot chromosome [30,34]. Further, the polytenized portion of the dot chromosome in *D. virilis *fails to stain with antibodies directed against HP1 [20] (Figure 1b).

Comparative genomics has been invaluable in discovering new functional and regulatory elements in the genomes of a cluster of yeast species, using *Saccharomyces cerevisiae *as the reference point [35]. We believe this comparative approach will be equally valuable as comparisons of *Drosophila *species become possible [36,37]. If the gene compositions of the dot chromosomes of *D. melanogaster *and *D. virilis *are similar, what other differences in the DNA sequence could lead to the apparent difference in higher-order chromatin structure? To address this question, we have generated a finished, clone-based sequence for a sample from the *D. virilis *dot chromosome and from the long chromosome arms; finished sequence leads to more accurate inferences about repetitive sequences [38]. By comparing similar regions of the two dot chromosomes, we show that while the overall repeat density of the dot chromosomes is similar, the density of DNA transposon remnants is significantly higher in *D. melanogaster *than in *D. virilis*; the difference is particularly striking for the *DINE-1 *elements and *1360 *elements, discussed above. These results, combined with recent findings about RNAi, lead us to suggest that the difference in chromatin packaging between the dot chromosomes of these two species of *Drosophila *could be a function of the density and distribution of a subclass of repetitive elements.

## Results

### Immunofluorescent staining indicates that the *D. virilis *dot chromosome is largely euchromatic, in contrast to the heterochromatic *D. melanogaster *dot chromosome

The dot chromosome of *D. melanogaster *is largely heterochromatic, with some interspersed domains of euchromatin [24]. Immunofluorescent staining of *D. melanogaster *polytene chromosomes using HP1 antibody shows a banded pattern on the dot chromosome. Many species in the *Drosophila *genus closely related to *D. melanogaster *share this staining pattern, including *D. simulans*, *D. yakuba*, and *D. pseudoobscura *(data not shown). In *D. melanogaster*, staining with an antibody against histone H3 methylated at lysine 9 (anti-H3K9me) coincides with the HP1 staining, at a level slightly less than seen in the pericentric heterochromatin [21] (Figure 1a). In contrast, the dot chromosome of *D. virilis *does not stain with either anti-HP1 or anti-H3K9me (Figure 1b), supporting the inference that the banded portion of the dot chromosome of *D. virilis *is generally euchromatic.

### Identification of fosmids from the dot chromosome of *D. virilis*

**Figure 2:**
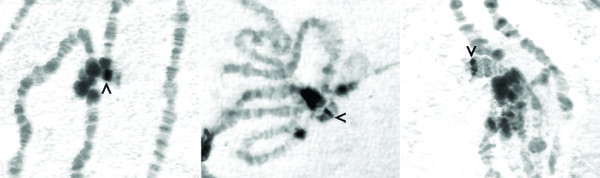
*In situ *hybridizations of fosmids to *D. virilis *polytene chromosomes. Fosmid DNA was labeled and used for *in situ *hybridization on denatured polytene chromosomes from *D. virilis*. Three examples are shown (left to right: contigs 106, 72, 113) demonstrating hybridization to a specific band on the dot chromosome (arrowhead). In some cases, signal is associated with the chromocenter, presumably due to repetitive sequences shared with the band on the dot. *In situ *hybridizations were performed with at least one fosmid from every contig from the dot chromosome with similar results (data not shown). See Table 1 for the chromosome locations of the other fosmids.

The chromosomes of *D. virilis *tend to map to corresponding portions of the chromosomes of *D. melanogaster *[39]. We compared the recently posted genomic sequence for *D. pseudoobscura *[37,40] with the *D. melanogaster *dot chromosome genes to look for regions of sufficient sequence similarity to act as conserved hybridization probes. The desired probes (see Materials and methods) were radiolabeled and used to screen a *D. virilis *genomic library (BDVIF01 fosmids, Tucson strain 15010-1001.10, available spotted on a single filter) at low stringency. Positive clones were verified and characterized by *in situ *hybridizations to the polytene chromosomes from third instar larval salivary glands of *D. virilis*. Sample results are shown in Figure 2. Eleven fosmids were recovered with homology to the dot chromosome of *D. virilis*, and seven fosmids were recovered with homology to the major chromosome arms. Based on the *in situ *hybridization results, the order of the fosmid clones on the dot chromosome is as follows: contigs 30, 103, and 106 appear to cluster near the centromere; contigs 67, 72, and 91 are in the middle of the chromosome; and contigs 50 and 113 hybridize near the telomere. There is also a minor signal with the contig 30 probe near the telomere; this may be the result of a repetitive element present in multiple regions in the chromosome.

### Fosmid sequencing and annotation

The 18 fosmids recovered from the screen were sequenced in collaboration with the Genome Sequencing Center at Washington University School of Medicine. Plasmid subclone libraries were prepared and approximately 600 subclones from each fosmid were end sequenced. The sequences were assembled and finished to high quality by Washington University undergraduate students in the Bio 4342 'Research Explorations in Genomics' course, using *phred*, *phrap*, and *consed *[41-43]. Finished sequences had an estimated error rate of less than 0.01%, and showed *in silico *restriction digests that matched digests obtained from the starting fosmid with a minimum of two enzymes. Students annotated the finished sequences by looking for genes, repetitive elements, and other features as described in Materials and methods. Four pairs of fosmids have significant sequence overlap; each pair was collapsed into a single contig of non-redundant sequence (contigs 30, 50, 67, and 80).

**Figure 3:**
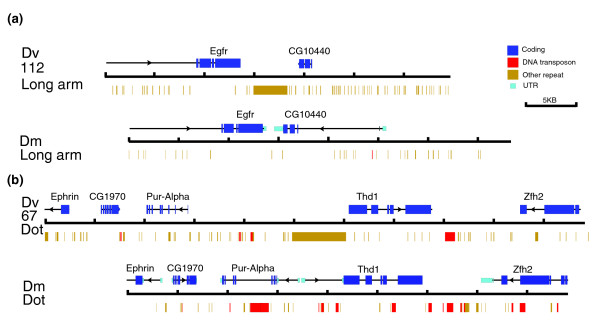
Map for two sample contigs from *D. virilis *(Dv) in comparison with homologous regions of the *D. melanogaster *(Dm) genome. Shown are two contigs from *D. virilis *with the corresponding regions from *D. melanogaster*. Coding sequences (dark blue boxes) are indicated above each diagram. In the case of *D. melanogaster*, the thick dark blue bar indicates open reading frames (ORFs), and the thin aqua bar indicates UTRs; only ORFs are identified for *D. virilis*. Repeat sequences are shown below: red boxes are DNA transposon fragments, while other repetitive elements are represented as yellow boxes. **(a) **Contig 112 represents a clone from one of the large chromosomes of *D. virilis*. While the orientations of *Egfr *and *CG10440 *are the same with respect to each other, there is a large tandem repeat between the two genes in *D. virilis*, but not in *D. melanogaster*. **(b) **Contig 67 represents a clone from the dot chromosome of *D. virilis*. The structure of the genomic region is similar to the corresponding region in *D. melanogaster*, but there is more intergenic space in *D. virilis*, whereas in *D. melanogaster*, there are more transposable elements in the introns. All of the fosmids described here with homologous regions in *D. melanogaster *have been annotated in a similar manner; the maps are available in the Additional data files. Scale: one division equals 5 kb.

Initial annotation focused on gene finding. *D. virilis *is evolutionarily close enough to *D. melanogaster *that the protein coding regions are well conserved. Gene prediction algorithms and local alignment search tools (such as GENSCAN and BLAST; see Materials and methods) were used to annotate genes and determine intron-exon boundaries. In most cases, it was possible to identify the entire coding region of the gene, but the high level of sequence divergence made defining untranslated regions impossible [36]. Comparison of the *D. virilis *contigs with homologous regions of the *D. melanogaster *dot chromosome identified specific regions where synteny has been maintained, as well as those regions where inversions have occurred. Figure 3 shows a comparison of two *D. virilis *contigs with the homologous regions from the *D. melanogaster *chromosomes. Detailed annotation results and comparisons between the other individual *D. virilis *fosmids and their homologous regions in *D. melanogaster *are available as Additional data file 1 (dot chromosome sequences) and Additional data file 2 (non-dot chromosome sequences). Note that the strain of *D. virilis *used here is a different strain from that recently sequenced (by Agencourt Bioscience Corporation, Beverly, MA, USA). The two strains differ by about 1% base substitutions, with numerous insertions or deletions (indels), but show similar organization at the gene level (CDS, unpublished observation). The clone-based sequencing used here results in more accurate inferences in regions that are highly repetitive; the sequences most likely to be missed in whole genome shotgun techniques are the repeats [38].

**Figure 4:**
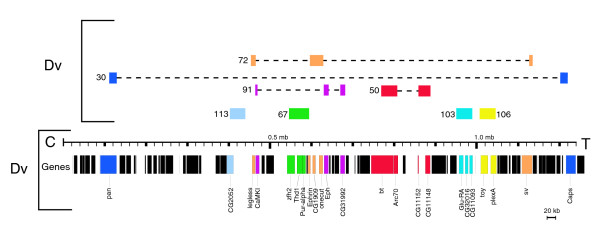
Map of the *D. virilis *(Dv) dot chromosome contigs in relation to the dot chromosome of *D. melanogaster *(Dm). Shown at the bottom is a map of the genes on the *D. melanogaster *dot chromosome. Colored bars with labels represent genes for which we have identified a (complete or partial) homologue in the *D. virilis *fosmids sequenced. Colored boxes above the scale bar are schematic (not to scale) representations of the *D. virilis *contigs. Immediately above the scale bar is a representation of those sequenced contigs that contain syntenic regions from *D. virilis*, where genes are in the same order and orientation as in *D. melanogaster*. In the uppermost portion of the figure are the contigs mapping to the *D. virilis *dot chromosome that are rearranged with respect to the *D. melanogaster *dot chromosome. Boxes are color-coded to represent the genes present in the contig, with dashed lines connecting to show the extent of rearrangement. Notably, contig 30 contains both *pan *and *Caps*, which lie on opposite sides of the banded portion of the *D. melanogaster *dot chromosome.

Table 1 shows all contigs sequenced, giving their total sizes, listing annotated genes, and providing clone names (BACPAC Center). *In situ *hybridization results identified the fosmids as either on the dot chromosome or on a major *D. virilis *chromosome. In parentheses following each gene is the chromosome position of the gene in the genome of *D. melanogaster*. Figure 4 maps the contigs from the dot chromosome of *D. virilis *to the dot chromosome of *D. melanogaster *based on the presence of orthologous genes. Three of the contigs (67, 106, and 113) are completely syntenic with respect to the *D. melanogaster *dot chromosome. One contig, 103, is completely syntenic with respect to its genes from the dot chromosome, but also contains *CG5367*, a gene from the second chromosome of *D. melanogaster*. Four contigs (30, 72, 50, and 91) contain genes that are exclusively from the dot chromosome of *D. melanogaster *but show evidence of a high number of inversions with respect to the *D. melanogaster *chromosome. For example, contig 30 contains both *pan *and *Caps*, genes that come from opposite sides of the banded portion of the *D. melanogaster *dot chromosome. (This rearrangement was also observed in earlier studies [31].) Of the 28 genes identified in the *D. virilis *dot chromosome clones, only one lies elsewhere in the *D. melanogaster *genome. In the *D. virilis *contigs from major chromosomes, four (contigs 13, 112, 121, 122) are completely syntenic compared to homologous gene regions from *D. melanogaster*, and two (contigs 11 and 80) show inversions within the chromosomes. Only one major chromosome contig (80) contains a gene that is found on the dot chromosome in *D. melanogaster*. Contig 80 maps to a major arm of *D. virilis; *it contains *D. melanogaster *dot chromosome gene *CG1732 *flanked by several genes from *D. melanogaster *chromosome 3. In total, the fosmids sequenced represent 372,650 bp of sequence from the dot chromosome of *D. virilis *and 273,110 bp of sequence from the major chromosomes. *D. virilis *contigs 72 and 91 from the dot chromosome and 11 and 80 from the major arms showed so much rearrangement that it was impossible to define precise homologous area(s) from *D. melanogaster*. These contigs were not used in comparisons for intron size, percent DNA transcribed, or in any of the repeat density calculations. Maps representing locations and sizes of genes and repeats in each contig are available in Additional data files 1 and 2.

### Average intron size and percent DNA transcribed

**Figure 5:**
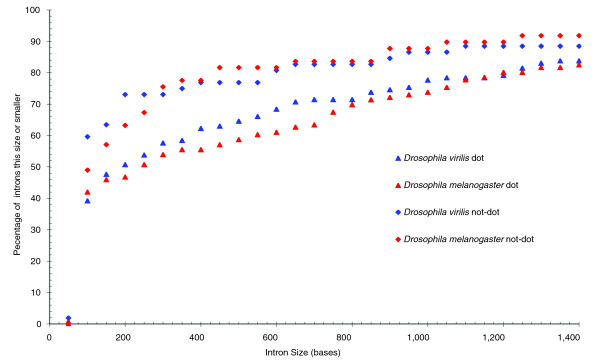
Distribution of intron sizes in *D. virilis *compared to *D. melanogaster*. Introns from all *D. virilis *and *D. melanogaster *genes in the contigs studied were separated into groups based on size. The number on the *x *axis represents the minimal intron size; an intron is counted in that bin if it has that many bases or fewer. The *y *axis tallies the percent of total introns that fall into that bin. The two dot chromosomes have significantly similar intron size distributions, which differ significantly from those of the major chromosome arms.

While centromeric regions are rich in satellite DNA and relatively gene poor [3], gene density (defined as the number of genes per Mb) in the banded portion of the dot chromosome is similar to the major chromosomes of *D. melanogaster *[19] (66.5 genes/Mb for the dot and 74.6 genes/Mb for the major chromosomes for the regions analyzed here). This is also true for the regions of the *D. virilis *genome we have sequenced (62.2 genes/Mb for the dot and 67.3 genes/Mb for major chromosomes). Observation of those few heterochromatic genes that have been cloned and sequenced (for example, *light *[44]) suggests that these genes may have larger introns on average, and this has been reported for *D. melanogaster *dot chromosome genes [19]. Average intron size, defined as total intron length divided by total number of introns, is 448 bp (± 126 bp) for our sample from the major *D. virilis *chromosomes and 405 bp (± 110 bp) for the corresponding regions of *D. melanogaster*. *D. virilis *dot chromosome genes in our sample have an average intron length of 890 bp (± 179 bp); in homologous regions of the *D. melanogaster *genome, it is 859 bp (± 115 bp). Figure 5 shows a graph that compares the intron size cumulative distribution functions of the dot chromosomes with the major chromosomes. Due to the non-normal distribution of intron sizes, the non-parametric Kolmogorov-Smirnov (KS) test is used to evaluate the statistical significance in the pairwise comparisons. The KS test indicates that the difference in the distribution of intron sizes between the two dot chromosomes is not statistically significant (D = 0.1237, *p* = 0.2816). However, the distribution of intron sizes for the dot chromosomes is significantly different from those for the major chromosomes for both species (D = 0.223, *p* = 0.0496 and D = 0.245, *p* = 0.0291 for *D. virilis *and *D. melanogaster*, respectively).

Percent DNA transcribed, defined as primary transcript length over total sequence length, is more similar between the homologous chromosomes than between the dot chromosomes and the major chromosomes. (In this instance, 5' and 3' untranslated regions (UTRs) were not scored in calculations of percent DNA transcribed, as these regions could not be identified in the putative *D. virilis *genes.) The sequenced regions of the *D. virilis *and comparable regions of the *D. melanogaster *dot chromosomes have transcript densities of 58.7% and 51.0%, respectively, while transcript densities of the major chromosomes are 22.2% for *D. virilis *and 25.9% for *D. melanogaster*. The difference in percent DNA transcribed between the dot and non-dot contigs reflects the larger average size of introns in the dot chromosome genes.

### (dC-dA)·(dG-dT) dinucleotide repeat frequency

One marker of euchromatin is the presence of abundant (dC-dA)·(dG-dT) dinucleotide repeats, also known as CA/GT repeats. *In situ *hybridization shows that these repeats are widely distributed in euchromatin, but that the dot chromosome of *D. melanogaster *has a much lower density of these repeats [5]. The dot chromosome of *D. virilis *has a CA/GT repeat frequency similar to its major autosomes, as shown by *in situ *hybridization [33]. Dinucleotide repeat analysis of the sequences from the *D. virilis *fosmids in comparison with the homologous regions of the *D. melanogaster *genome supports the *in situ *hybridization results. The fosmids from the dot chromosome of *D. virilis *have CA/GT repeats with an average length of 36 bp and a total density of 0.15%. Regions of the *D. melanogaster *dot chromosome homologous to these fosmids have only one CA/GT repeat, which is 21 bp long, giving a total CA/GT density of 0.0069%. In the *D. virilis *clones mapping to major chromosomes, 0.96% of the DNA is made up of CA/GT, with the average repeat being 32 bp long. In homologous regions of the *D. melanogaster *genome, 0.32% of the DNA is CA/GT, with the average length of dinucleotide regions being 24 bp. Thus, while the *D. virilis *dot chromosome has a lower level of CA/GT than the major chromosome arms (about six-fold less than *D. virilis *and about two-fold less than *D. melanogaster*), it has a approximately 20-fold higher level of this repeat than is found in the dot chromosome of *D. melanogaster*.

### Repeat analysis

Initial analysis of known repetitive elements in the *D. virilis *contigs was performed using RepeatMasker [45]. RepBase 8.12 [46,47] contains previously characterized repeats from the *D. virilis *species group. As a simple initial approach we searched for *de novo *repeats by comparing the fosmid sequences to each other, looking for regions of high similarity by BLASTN [48]. Most apparently novel repeated sequences identified by this technique were immediately adjacent to known repeats identified by RepeatMasker and were, therefore, assumed to be unmasked extensions of those repeats. A few novel repeats were identified that were not similar to any other known repetitive element, expressed sequence tag (EST), or protein sequence. Using this simple technique, novel repeats constituted less than 1% of the total repetitive DNA; however, given the small size of our dataset (0.65 Mb) it is possible that repetitive elements could be missed.

**Figure 6:**
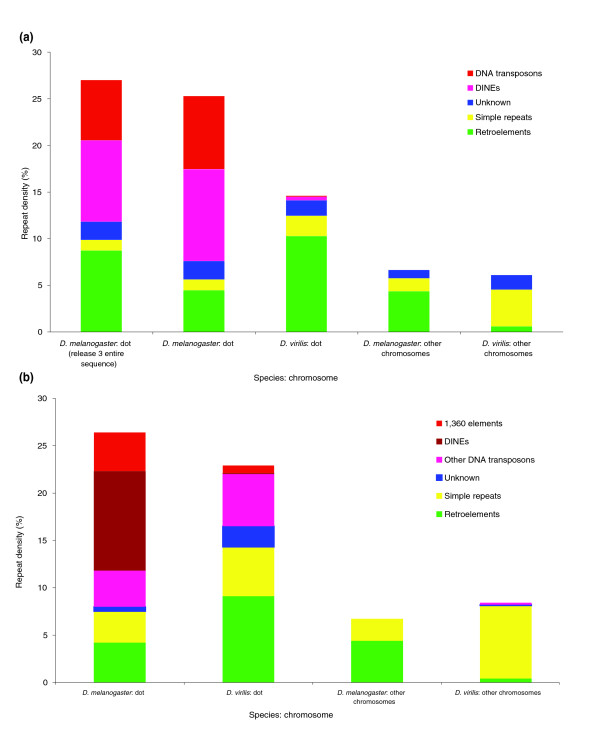
Repeat analysis of *D. virilis *contigs compared to the *D. melanogaster *genome. The repeat density, defined as the percentage of total sequence (in base-pairs) that has been annotated as repetitive has been calculated using the *D. virilis *fosmid sequence obtained in this study and homologous regions from *D. melanogaster *(see Materials and methods). *D. melanogaster *and *D. virilis *have a very similar low repeat density on the major chromosome arms, and a similar but much higher repeat density on the dot chromosomes. **(a) **Percent repeat for each type identified by RepeatMasker using RebBase 8.12 with additional repeats identified in a BLASTN all-by-all comparison of the fosmid sequences presented here. **(b) **Percent repeat for each type identified by RepeatMasker using the Superlibrary (see text for description). The dot chromosome of *D. melanogaster *has about three times more DNA transposon sequence than does the *D. virilis *dot chromosome. 'Unknown' repeats are those from both RebBase 8.12 and the *D. virilis *PILER-DF library that have not been classified as to type.

Figure 6a shows the repeat density of different classes of repetitive elements in the *D. virilis *contigs and the comparable regions of the *D. melanogaster *genome using RepeatMasker/RepBase (*Drosophila *default parameters) plus this simple *de novo *BLASTN technique. While there is some variation in repeat density between the contigs of a given region (dot chromosome or major chromosome), the totals appear to represent an average value of the contigs studied. Using this analysis, the overall repeat density of the *D. virilis *dot chromosome contigs is 14.6%; the average of the individual repeat densities is 15.4% ± 7.9%. The overall repeat density of the homologous *D. melanogaster *regions is 25.3%; the average of the individual repeat densities is 24.7% ± 5.4%. Fosmids from the dot chromosome of *D. melanogaster *show a consistently higher density of DNA transposons and *DINE-1 *elements than do the fosmids from the dot chromosome of *D. virilis*. Comparison of the sample from the dot chromosome of *D. melanogaster *analyzed here to the entire banded portion of the dot chromosome (using RepeatMasker and RepBase 8.12) shows very similar results (Figure 6a). In contrast, the euchromatic arms of the large chromosomes of *D. melanogaster *and *D. virilis *have similar repeat densities, with approximately 6% of the sequence classified as repetitive. (Quesneville *et al*. [49] estimate the total repeat density of *D. melanogaster *to be 5.3%.) Other repeat types differed between the two species as well. In our sample from these chromosome arms, *D. virilis *has more simple repeats and *D. melanogaster *has more retroelements. Overall, these results suggest that both the higher repeat density and the overrepresentation of DNA transposons contribute to heterochromatin formation on the *D. melanogaster *dot chromosome. However, because *D. virilis *is not as well studied as *D. melanogaster*, it is possible that this approach misses some uncharacterized repeats. To address this issue, we undertook several different strategies.

Recent investigations have developed multiple search tools for *de novo *identification of novel repetitive sequences in genome assemblies [50,51]. Using such tools, we created a 'Superlibrary' in which we added sequences from species-specific libraries from both *D. melanogaster *and *D. virilis *to the RebBase 8.12 Drosophila transposable element (TE) library to generate a library with as little bias as possible. The additional repeats came from three sources. Two novel repetitive elements that were identified in *D. melanogaster *using the PILER-TR program were added [50]. We also added a complete set of 66 elements from *D. virilis *identified by PILER-DF analysis (C Smith and G Karpen, personal communication) of the posted *D. virilis *whole genome assembly [52]. Finally, a recently identified sequence of *DINE-1 *from *D. yakuba *was added [53].

All of the *D. virilis *and *D. melanogaster *sequences used in this study were then analyzed for repetitive DNA using RepeatMasker with this Superlibrary. This approach identified a total repeat density of the *D. virilis *contigs from the dot chromosome of 22.8%, while homologous regions of the *D. melanogaster *dot chromosome have 26.5% repetitive DNA (Figure 6b). Using the same Superlibrary, the segments from the major chromosomes of *D. virilis *have a total repeat density of 8.4%, compared to *D. melanogaster *major chromosomes, which have a density of 6.8%. This analysis shows that the overall density of repeats on the *D. virilis *and *D. melanogaster *dot chromosome fosmids is similar, and significantly higher than the density of repeats on the major chromosomes from either species. Other analysis techniques used to assess the difference between the *D. virilis *and *D. melanogaster *sequences, including a TBLASTX comparison using a RebBase 8.12 library from which invertebrate sequences had been removed [49,54], and a Repeat Scout library assembly [51], also showed little difference in the total amount of repetitive sequence found in the *D. virilis *and *D. melanogaster *dot sequences (not shown). Thus, all of the follow-up techniques applied indicate that the sequences from the dot chromosomes of both *D. virilis *and *D. melanogaster *are enriched for repetitive sequences compared to the sequences derived from the major chromosomes of both species. The analysis of each contig as well as the total representation of each type of repeat is presented in Table 2 and in Figure 6b. The contrast between the results shown in Figure 6a and those shown in Figure 6b illustrates the problem posed by biased repeat libraries, an issue that must be carefully considered in studies of this type. The observation that three different analyses (discussed above) support the results shown in Figure 6b lends confidence to the conclusions derived here.

While the overall density of repetitious elements is similar, there is a major difference in the density of DNA transposons (Table 2). Of the *D. melanogaster *dot chromosome DNA from our sample, 18.6% consists of remnants of DNA transposons, including sequences from *1360 *elements, *P *elements (artifacts and related fragments), *Tc1 *elements and *DINE-1*. Only 6.4% of these regions from the dot chromosome of *D. virilis *consists of remnants of DNA transposons, about a three-fold reduction. The bulk of the repetitive sequence in the *D. virilis *dot fosmids tentatively classified as DNA transposons are the dvir.16.2 centroid and the dvir.16.17 centroid, sequences identified in the PILER-DF analysis. Table 3 shows the repeat element and class of the most common repeats in the *D. virilis *and *D. melanogaster *dot chromosome contigs studied here, as identified by RepeatMasker/Superlibrary. DNA transposon families are preferentially represented in *D. melanogaster*, while retroelements (LINEs and LTRs) are more common in *D. virilis*. Examination of the quantitative results in Table 2 suggests that the dot chromosome of *D. virilis *has an increase in retroelements (9.1%) in comparison with homologous regions of *D. melanogaster *(4.2%). However, this difference appears to be due to sample bias, as RepeatMasker/RebBase 8.12 classifies 8.7% of the whole *D. melanogaster *dot chromosome as retroelements.

*DINE-1*, also known as *DNAREP-1 *or *INE-1*, is a repetitive element that is very common in the genome of *D. melanogaster *[55]. The density of *DINE-1 *elements is especially high on the dot chromosome of *D. melanogaster*, more so than on the major chromosome arms or on the dot chromosome of *D. virilis *[17,56]. Using our Superlibrary and repeat identification process, RepeatMasker identifies 0.1% of the *D. virilis *contigs as sequences with significant similarity to *DINE-1 *elements, while in the homologous regions of the *D. melanogaster *dot the density is 10.5%. (The entire *D. melanogaster *dot has a 9.2% incidence of *DINE-1 *elements, assessed using RepeatMasker/Superlibrary.) There has been considerable debate as to the origin of *DINE-1 *elements [56,57]. Kapitonov and Jurka [57] have recently suggested that *DINE-1 *is a retrotransposon based on homology to a *D. virilis Penelope *GenBank accession, but sequences with homology to *DINE-1 *in this accession fall outside of the canonical *Penelope *sequence [58] (C Bergman, personal communication). Analysis of *DINE-1 *elements in *D. yakuba *suggests a relatively recent burst of transposition in that species. A consensus sequence based on these recent *DINE-1 *elements contains no long terminal repeats nor a poly-A tail (suggestive of a retroelement), but does have a terminal 12 bp perfect repeat, a characteristic of transposons [53]. Thus, while we have provided separate statistics for this class, we consider *DINE-1 *elements to be DNA transposon remnants. Separate statistics are also provided in Table 2 for the *1360 *DNA transposon fragments, as this class is of particular interest as a potential target for heterochromatin formation, as discussed above. Again, this family is significantly enriched in *D. melanogaster *dot chromosome fosmids, making up 4.1% of the sample DNA, in comparison to 0.8% in *D. virilis *dot chromosome fosmids and 0% in the samples from the major chromosome arms.

Transposable elements are much more prevalent in the introns of heterochromatic genes than in the introns of euchromatic genes [4]; this may contribute to the evolution and structure of genes in heterochromatin. Maintaining a focus on total repeat density (and not repeat type), we analyzed the introns of all of the contigs with a repeat database generated by combining the RepeatScout output from both the *D. melanogaster *and the *D. virilis *whole genome assemblies. Using RepeatMasker with this library (omitting low complexity and simple repeats), one finds that introns of the *D. virilis *dot chromosome genes studied here contain 27.0% repetitive elements, while in homologous regions of the *D. melanogaster *dot, 33.1 % of the introns are made up of repetitive elements. Analysis of the contigs from the major chromosomes of *D. virilis *and the homologous regions from *D. melanogaster *did not find any recognizable transposable elements in the introns. Thus the two dot chromosomes are in this respect more similar to each other than they are to the major chromosomes from either species.

Comparing Figures 6a and 6b, it is apparent that the two repeat-finding strategies represented gave very different results. *D. melanogaster *and *D. virilis *are fairly close together phylogenetically, but use of the previously defined RepBase library, which has good representation of *D. melanogaster *repeats, was insufficient to find all of the *D. virilis *repeats, particularly on the dot chromosome. This result stresses the importance of using techniques such as PILER to find species-specific repeats as new species are sequenced, even when repeat sequences are available from a well-studied nearby species. Relying on existing repeat databases can lead to erroneously low estimates of repeat content.

## Discussion

### The dot chromosomes of *D. melanogaster *and *D. virilis *differ in the density of DNA transposons

While one of the conspicuous characteristics of pericentric heterochromatin is a low gene density, previous sequence analysis has shown that the heterochromatic *D. melanogaster *dot chromosome resembles euchromatic domains in this regard, having a gene density (number of genes per Mb) similar to the long arms of the major autosomes [12,19]. Interestingly, the *D. melanogaster *dot chromosome does have an approximately two-fold higher percentage of DNA transcribed (percentage of DNA between the start sites and stop sites for transcription) than the major chromosomes, due primarily to longer introns in the dot chromosome genes. Introns of dot chromosome genes of both species examined here were longer than introns from the major chromosomes (Figure 5), apparently reflecting the higher repeat content of the dot chromosomes (see above). Thus, the heterochromatic *D. melanogaster *and the euchromatic *D. virilis *dot chromosomes are very similar to each other in gene density, percent DNA transcribed, and gene/intron size, suggesting that these parameters are not critical in determining chromatin packaging decisions.

Total repeat density (percentage of the DNA in repetitive sequences) for the *D. virilis *dot chromosome fosmids has a value of 22.8%, while the homologous regions of the dot chromosome of *D. melanogaster *have a total density of 26.5%. Kaminker *et al*. [18] analyzed the distribution of transposable elements (not including simple or tandem repeats) in the *D. melanogaster *genome. This analysis indicated that the number of repetitive elements per Mb is five to ten times higher on the dot chromosome than in the rest of the sequenced genome, which includes very little heterochromatin. The repeat analysis of our region of study agrees with the whole chromosome results from Kaminker *et al*. [18] in that the level of repetitive elements (predominantly partial or dead TEs) shows a large difference (about three- to four-fold) between the dot chromosomes and the major chromosomes. Our analysis reported here shows that there is only a small difference in the total repeat density on the heterochromatic *D. melanogaster *dot chromosome and on the euchromatic *D. virilis *dot chromosome. This finding suggests that the higher density of repetitive elements probably does not play a deciding role in driving the heterochromatic packaging of the dot chromosome in *D. melanogaster*. This does not preclude the possibility that high repeat densities are a necessary precondition for heterochromatin formation, but argues that a high repeat density is not sufficient in and of itself to drive formation of heterochromatin.

This analysis rather focuses attention on the high level of DNA transposons found in the *D. melanogaster *dot chromosome, but lacking in the *D. virilis *dot chromosome. Prominent elements of this type in *D. melanogaster *include *1360 *(aka *hoppel *or PROTOP_A) and *DINE-1*. It has previously been suggested that *DINE-1 *might contribute to heterochromatin packaging on the dot chromosome of *D. melanogaster *[17,56]. In our computational analysis, we found that sequences homologous to *DINE-1 *were also present on the dot chromosome of *D. virilis*, but at a much lower concentration (0.1%) compared to *D. melanogaster *(10.5%), in agreement with the *in situ *hybridization analysis previously reported [56]. Our computer homology searches and analysis by others [57] indicates that portions of the *DINE-1 *element found in RebBase 8.12 show high similarity to a genomic fragment containing *Penelope *elements from *D. virilis*, but this similarity falls outside of the region defined by Evgen'ev *et al*. to be required for *Penelope *activity [53,58] (C Bergman, personal communication). Thus, in our analysis, *DINE-1 *has been treated as a DNA transposon type, but has been reported separately in Figure 6 for clarity. *DINE-1 *is absent from the major chromosome arms in this sample. In contrast, *Penelope *elements are very common in the dot chromosome (approximately 9%), and are present in the major chromosomes of *D. virilis *(0.4%). Retrotransposons such as *Penelope *are important in determining chromosome rearrangements [59], but have not been associated with heterochromatin formation.

It has been suggested that the buildup of repetitive elements on the dot chromosome may be due in part to the lack of recombination [19,60]. However, we find an overabundance of repetitive sequences on the dot chromosomes of both *D. melanogaster *and *D. virilis*. Recombination does occur on the dot chromosome of *D. virilis*, albeit at a lower rate [30]. This observation suggests that there may be a selective advantage in maintaining a higher than average density of repetitive sequences (and larger than average genes) in this small chromosome, regardless of the chromatin packaging status.

### DNA transposons may be targets for heterochromatin formation

Work by Sun *et al*. [24] suggests a particular DNA transposon that may act as an initiator of heterochromatin formation on the dot chromosome of *D. melanogaster*. If a *white *reporter *P *element insertion site is within 10 kb of a *1360 *element on the dot chromosome, there is a high probability of a variegating phenotype [24]. Hence remnants of this DNA transposon may serve as a *cis*-acting determinant of heterochromatin formation on the dot chromosome of *D. melanogaster*, presumably acting as targets of an RNAi-directed process [26] analogous to that reported in *S. pombe *[1].

*1360 *elements are fragments of a DNA transposon that has been recognized in many studies to have a high concentration on the dot chromosome and in the pericentric heterochromatin [17,19]. Coelho *et al*. [60] studied *1360 *in many different strains of *D. melanogaster *and found that the association with heterochromatin is very consistent, again suggesting that *1360 *elements play an important role in the structure and function of heterochromatin. Given a lack of introns, it has been suggested that *1360 *elements are derivatives of a retrotranscription event [61], but because this element has terminal inverted repeats at its ends and encodes a transposase with similarity to the *P *enzyme [57,61], it is likely to function as a DNA transposon. *1360 *may be a very recent invader of the *D. melanogaster *genome [57]; some differences in insertion sites are observed in different stocks of *D. melanogaster *[24,60]. The origin of RNAi is thought to be as a silencing mechanism for retroviral genome invaders or transposons with multiple exact copies. Thus, it is possible that recent invaders are most likely to be targets of RNAi-induced heterochromatin formation.

Some regions of the *D. melanogaster *dot chromosome have significantly lower density of DNA transposons other than *DINE-1 *than the chromosome as a whole, particularly contigs 30.1, 106, and 67 (Table 2). The density of *DINE-1 *elements in these contigs is similar to that in the rest of the dot chromosome, but the level of other DNA transposons is less than 2%. Interestingly, these regions appear to be euchromatic domains of the dot chromosome of *D. melanogaster *as shown by a *white *reporter. The region around contig 30.1 is within 25 kb of an insertion site for a *P *element with full red eye expression, and the region around contig 67 is within 20 kb of six *P *elements with full red eye expression [24]. This suggests that the local density of repetitive elements other than *DINE-1 *may be important in driving changes in chromatin structure, or that another factor might be countering the influence of *DINE-1 *in these regions.

Why might DNA transposons be a preferred target for heterochromatin formation? DNA transposons contain inverted repeats at each end that facilitate their mobilization within the genome; these could intrinsically lead to dsRNA if both ends are transcribed. This has been reported in *C. elegans *[62], but we are not aware of any similar reports for *D. melanogaster*. Using a *P *element reporter, Dorer and Henikoff [63] showed that DNA transposon mobilization events can lead to tandem and inverse duplications of the *P *element. These tandem arrays can lead to heterochromatin formation and silencing of a reporter gene within the *P *element construct. It is also possible that an endogenous transposable element could be present as inverted copies in an intron; transcription could then produce hairpins that could be targeted by the RNAi machinery for degradation and result in heterochromatin-mediated transcriptional gene silencing. (Fragments of LINE elements in inverted orientations in the introns of mammalian genes have been found to be a source of miRNAs [64].) Thus, the mode of mobilization might generate configurations of DNA transposon sequence that make these elements a preferred target for RNAi-directed gene silencing and heterochromatin formation.

Our computational analysis has shown that inverted fragments of TEs can readily be found in introns in both species. Screening for inverted repeats (IRs; using RepeatMasker/RebBase 8.12) located within a single intron and within 100 bp of each other revealed 87 copies of inverted TEs (81 *DINE-1*, 2 *1360 *elements, 4 *S2_DM *elements) within the *D. melanogaster *genome and three (all *Penelope*) within our *D. virilis *fosmids. Some of these candidates are predicted to form stable hairpin structures by mfold [65]. Among these hairpin candidates are the *1360 *IR found in an intron of *Caps *on the *D. melanogaster *dot chromosome, and the *Penelope *IR found in an intron of *toy *on the *D. virilis *dot chromosome. Transcription through these loci could create hairpin structures that might be subsequently processed by the Drosha and Dicer machinery to produce short dsRNA, leading to initiation of heterochromatin formation. Hence the potential exists for both DNA transposons and retroelements to act as targets for RNAi-directed gene silencing and heterochromatin formation; why the former appears to be favored is not clear.

Empirically, the absence of heterochromatin formation on the *D. virilis *dot chromosome appears not to be related to a lower density of repetitive elements in this genomic domain, but may be a consequence of the low density of DNA tranposons. Gene density, percent DNA transcribed, and size of introns do not seem to be critical discriminatory factors. However, a higher frequency of CA/GT repeats on the *D. virilis *dot chromosome is associated with euchromatic chromatin packaging. No mechanism has been suggested to explain the significance of this correlation. Other differences in primary sequence and density of particular transposable elements between the dot chromosomes of these two species, as yet unidentified, could also play a role. Genomic data is forthcoming on many *Drosophila *species [52]. A comparison of several heterochromatic dot chromosomes with several euchromatic dot chromosomes will no doubt further elucidate the basis of heterochromatin formation in *Drosophila*.

## Materials and methods

### Chromosome staining

Immunofluorescent staining of polytene chromosomes from *D. melanogaster *(Oregon R) and *D. virilis *(Tucson strain 15010-1001.10) third instar larvae was carried out as described [66]. HP1 antibody was monoclonal mouse C1A9 antibody (cell supernatant) used at a dilution of 1:1. Rabbit antibody for histone H3 methylated at lysine 9 was from Upstate Biotechnology (Lake Placid, NY, USA) used at a dilution of 1:25. Secondary antibodies were labeled with Alexa fluor 488 (Molecular Probes, Eugene, OR, USA) for goat anti-mouse and Alexa fluor 594 (Molecular Probes) for goat anti-rabbit, both used at a dilution of 1:400.

### Identification of fosmids from the dot chromosome of *D. virilis*

Coding sequences from all genes from the dot chromosome of *D. melanogaster *were used in BLASTN [48] searches against the *D. pseudoobscura *NCBI trace archive [40]. Similar regions were entered into Block Maker [67] to find regions of highest conservation; PCR primers were designed around these regions using CODEHOP [68]. Criteria for inclusion were a length of at least 200 bp, with 80% homology between *D. melanogaster *and *D. pseudoobscura*, with screening to the *D. melanogaster *genome to ensure that the region did not contain any repetitive elements. PCR was performed using *D. melanogaster *genomic DNA as the template; the PCR products were subsequently labeled and used to probe the BDVIF01 library (of Tucson strain 15010-1001.10) originally described in Bergman *et al*. [36], now available spotted on a single filter from BACPAC [69]. Hybridizations and washes were performed at low stringency. Positive clones were verified using Southern blots, slot blots, and restriction mapping. Some of the recovered clones mapped to chromosomes other than the *D. virilis *dot; these appear to have been identified by cross hybridization resulting from low stringency hybridization and washing conditions.

### *In situ *hybridization of fosmids to polytene chromosomes

*In situ *hybridization probes were digoxygenin-dUTP labeled fosmid inserts. Hybridizations were performed on polytene chromosomes of third instar larvae of *D. virilis *(Tucson strain 15010-1001.10) as described by Casacuberta and Pardue [70].

### Sequencing of *D. virilis *fosmid clones

*D. virilis *fosmid DNA was prepared by streaking the glycerol stocks onto selective media agar plates, picking three isolated colonies and preparing a mini-prep of DNA from each. Mini-prep DNA was digested using *Hin*dIII and analyzed by agarose gel electrophoresis to compare restriction patterns to those obtained initially from the clones. Colonies verified by the restriction pattern were then inoculated into 200 ml of liquid media and grown in culture. Large-scale fosmid DNA isolation was performed from these cultures. The DNA was then sheared with the Hydroshear™ (manufactured by Genomic Solutions, Ann Arbor, MI, USA) to a nominal size range of 3 to 4 kb, end-repaired, and separated on an agarose sizing gel. The 3 to 4 kb band was excised from the gel and purified by phenol:chloroform extraction. The sheared insert DNA was subcloned into pZero2.1 (Invitrogen, Frederick, MD, USA), electroporated into DH10B cells, and the cells plated onto solid media. For each fosmid project, 768 subclones were picked into glycerol-containing media and archive stocks were grown for 24 hours at 37°C. Of these, 384 subclones were processed through the Genome Sequencing Center production sequencing pipeline, including magnetic bead-based DNA purification, dual end sequencing with Big Dye version 3.1 terminator chemistry (Applied Biosystems Inc., Foster City, CA, USA), and analysis on ABI 3730xl sequencers. Sequence assembly was performed using *phred*, *phrap*, and *consed *to design finishing strategies [42,43]. All fosmids were finished to the same quality standard as used for the human genome [71]. Sequences were confirmed by comparison of *in silico *digests of the finished sequence to restriction digest patterns of the purified fosmid DNA for at least two separate restriction enzymes. The nucleotide sequences and predicted protein sequences reported here have been submitted to GenBank (accession numbers DQ378280-DQ378293).

### Curation strategy

Gene sequences were initially identified by similarity in BLASTALL [48], comparing finished *D. virilis *fosmids to *D. melanogaster *protein, EST, cDNA and genomic sequences in GenBank. *D. virilis *fosmids were also compared to *D. pseudoobscura *genomic sequence [40] using BLASTN and TBLASTN [72]. Intron-exon boundaries were determined by visual inspection of coding matches, aided by Genscan [73,74], in conjunction with other annotated features. BLAT [75] was also used in this process, comparing intron-exon boundaries of predicted coding sequence across *D. melanogaster*, *D. yakuba *and *D. pseudoobscura*. Known protein-coding sequences, exon boundaries and intron sizes were obtained from Ensembl [76] and Flybase [13,77].

*D. virilis *retroelements, DNA transposons, low-complexity and simple repeat sequences were masked by RepeatMasker [45] using default parameters for *Drosophila *sequences. The RepeatMasker version used was from 03/06/04 with crossmatch version 0.990329 (RebBase update 8.12). Repeats unique to the *D. virilis *fosmids were predicted by BLASTN of each fosmid against a database of all the *D. virilis *sequences obtained, excluding regions that had significant *D. melanogaster *EST and protein BLASTALL matches.

The Superlibrary was created by combining repeats identified by the Drosophila TE library in RepBase 8.12, novel repeats identified using PILER-DF on the *D. virilis *dvirAra08 assembly [78] (C Smith and G Karpen, personal communication), novel *D. melanogaster *repeats identified using PILER-TR [50] and the *D. yakuba DINE-1 *element [53]. RepeatMasker was used with the Superlibrary to identify the portion of each fosmid or equivalent region that contained repetitive sequences.

RepeatScout (1.0.1) [51] was run with default parameters against the dmelWGS2 and dvirAra08 assemblies to generate the RepeatScout libraries for *D. melanogaster *and *D. virilis*. Tandem repeats and simple repeats in each RepeatScout library were removed using trf [79,80] and nseg [81,82]. The results were then combined to create a custom *Drosophila *RepeatScout library.

### Sequence comparison

Sequence comparisons were made of *D. virilis *fosmids with corresponding regions from the *D. melanogaster *(Release 3.2) genome, as determined by a set of reproducible guidelines involving syntenic features and equivalent flanking sequence. If a region of non-coding sequence from *D. virilis *was interrupted in the corresponding *D. melanogaster *sequence by an annotated gene, then only sequence up to that gene was included in the comparison. Where non-coding sequence flanked a corresponding gene in *D. melanogaster*, with no other identifying features that would indicate a loss of synteny, the extracted sequence extended an equal number of bases from the gene as seen in *D. virilis*. Several of the *D. virilis *fosmids (contigs 11, 72, 80, and 91) had no appreciable synteny with the *D. melanogaster *genome and were, therefore, not included in repeat comparisons between the two species. The gene density and intron size calculations take into account all fosmids.

### Kolmogorov-Smirnov test

The KS test is a non-parametric, distribution-free statistical test that can determine if two datasets differ significantly. It produces a D statistic that represents the maximum difference of the two distributions in an empirical cumulative distribution plot, allowing one to accept or reject a hypothesis that the datasets are from the same distribution. Statistical analysis was done using program R from the R Foundation for Statistical Computing [83].

### CA/GT repeat analysis

BLASTN [48] was used to search the contigs for CA/GT repeats. Blast databases containing either all of the *D. virilis *sequence obtained here or all of the corresponding regions from *D. melanogaster *were constructed using formatdb with default parameters. The databases were searched with a sequence of 100 (CA) repeats using BLASTALL default parameters except that low complexity filtering was turned off and the (E)xpect value was set to 0.1. The location of all hits was analyzed to remove any duplicate hits prior to assignment to either the dot or major chromosomes.

## Additional data files

The following additional data are available with the online version of this paper. Additional data file 1 provides maps of each fosmid from *D. virilis *and the homologous regions from *D. melanogaster *(if available) showing the genes and identified repetitive elements for dot chromosome sequences. Additional data file 2 provides maps of each fosmid from *D. virilis *and the homologous regions from *D. melanogaster *(if available) showing the genes and identified repetitive elements for non-dot chromosome sequences. Additional data file 3 provides a fasta file pre-formatted for use with RepeatMasker containing the PILER-DF identified repeats from the *D. virilis *assembly dvirAra08, the *D. yakuba DINE-1 *element, and the PILER-TR identified novel repeats from *D. melanogaster*, which were added to RepBase 8.12 *Drosophila *TE library to generate the Superlibrary used to analyze repeats.

## Supplementary Material

Additional data file 1 Maps of each fosmid from *Drosophila virilis *and the homologous regions from *Drosophila melanogaster *(if available) showing the genes and identified repetitive elements for dot chromosome sequences Click here for file

Additional data file 2 Maps of each fosmid from *D. virilis *and the homologous regions from *D. melanogaster *(if available) showing the genes and identified repetitive elements for non-dot chromosome sequences Click here for file

Additional data file 3 A fasta file pre-formatted for use with RepeatMasker containing the PILER-DF identified repeats from the *D. virilis *assembly dvirAra08, the *D. yakuba DINE-1 *element, and the PILER-TR identified novel repeats from *D. melanogaster*, which were added to RepBase 8.12 *Drosophila *TE library to generate the Superlibrary used to analyze repeats Click here for file

## Figures and Tables

**Table 1:** Annotation of the *D. virilis *contigs

Contig	BACPAC	Genes	Size (bp)	Repeat analysis
*D. virilis *dot chromosome fosmids				
30	15E14, 12E24	*pan *(4), *CG32005 *(4), *Caps *(4)	61,074	Yes
103	44I5	*CG5367 *(2L), *CG11093 *(4), *CG32016 *(4), *Glu-RA *(4)	39,850	Yes
106	39O6	*toy *(4), *plexA *(4)	40,734	Yes
67	23A13, 15G13	*Ephrin *(4), *CG1970 *(4), *Pur-alpha *(4), *Thd1 *(4), *zfh2 *(4)	54,154	Yes
72	3G18	*sv *(4), *lgs *(4), *onecut *(4), *CG1909 *(4), *Ephrin *(4)	43,948	No
91	42I6	predicted gene, *CG31992 *(4), *Eph *(4), *CaMKI *(4)	39,292	No
50	38M22, 34I22	*bt *(4), *Arc70 *(4), *CG11148 *(4), C *G11152 *(4)	56,333	Yes
113	47B4	*CG2052 *(4)	37,265	Yes
				
*D. virilis *fosmids from major chromosomes				
11	43O10	*CG32521 *(X), *Tim13 *(3L)	40,809	No
13	26E5	pseudogene, *CG31337 *(3R)	40,479	Yes
80	22L1, 42E12	*CG14129 *(3L), *CG5917 *(3L), *CG1732 *(4), *CG14130 *(3L), *CG9384 *(3L), *Trl *(3L), *CG9343 *(3L), *ome *(3L)	68,774	No
112	10J19	*CG10440 *(2R), *Egfr *(2R)	34,783	Yes
121	18G4	*CG17267 *(3R), *cdc2c *(3R), *Oamb *(3R)	47,154	Yes
122	36E24	*Syn *(3R), *CG12814 *(3R), *Best1 *(3R), *CG6995 *(3R)	41,111	Yes

The table lists contigs sequenced from *D. virilis*. The top section lists contigs from the dot chromosome of *D. virilis *in approximate order on the chromosome from centromere to telomere (as determined by *in situ *hybridization). The bottom section lists contigs from major chromosomes of *D. virilis *in an arbitrary order. The contig name is followed by the number(s) of the fosmid clone(s) sequenced (BACPAC Center at CHORI [69]). Genes are listed in the order in which they occur in the contig, with the number in parentheses representing the chromosome in which the homologous gene is found in the *D. melanogaster *genome. The total size of the contig is given; the final column indicates whether the contig was used in the repeat analysis (see Materials and methods).

**Table 2:** Repeat analysis of individual contigs from *D. virilis *compared to *D. melanogaster*

Contig	Size (bp)	*1360 *%	*DINE-1 *%	DNA %	Retro %	Simple %	Unk %	Total %
*Drosophila virilis *dot chromosome contigs								
30	61,074	1.9	0.0	5.6	15.1	5.6	1.8	30.1
50	56,333	0.0	0.0	3.7	0.0	2.8	5.3	11.9
103	39,850	0.0	0.0	7.8	5.6	5.7	4.3	23.3
106	40,734	2.6	0.0	7.4	4.0	5.5	0.6	20.1
67	54,154	0.0	0.1	3.8	10.3	6.0	0.1	20.3
113	37,265	0.0	0.2	5.8	20.5	6.2	0.3	33.0
**Totals**	**289,410**	**0.8**	**0.1**	**5.5**	**9.1**	**5.2**	**2.2**	**22.8**
72	43,948	0.3	0.4	9.7	1.4	10.5	1.1	23.4
91	39,292	0.0	0.0	0.2	9.2	6.8	0.5	16.7
								
*Drosophila melanogaster *dot chromosome contigs								
30.1	40,013	2.0	6.0	0.0	3.2	6.9	0.1	18.2
30.2	23,681	14.4	6.7	0.0	25.6	2.9	4.6	54.3
50.1	43,617	10.0	4.2	5.4	0.0	2.4	0.0	22.0
50.2	34,148	4.8	9.3	12.1	4.7	2.9	0.2	34.0
103	40,295	5.4	14.6	6.1	0.7	2.7	0.2	29.8
106	38,285	0.0	21.8	0.8	3.2	2.0	0.3	28.1
67	44,243	0.1	8.1	4.6	0.0	3.7	0.2	16.7
113	36,538	0.0	13.0	0.1	6.2	2.7	0.4	22.4
**Totals**	**300,820**	**4.1**	**10.5**	**3.8**	**4.2**	**3.3**	**0.5**	**26.5**
*Entire 4*^th^	*1,237,870*	*3.8*	*9.2*	*3.0*	*9.5*	*2.9*	*0.0*	*28.4*
								
*Drosophila virilis *contigs from major chromosomes								
13	40,479	0.0	0.0	0.4	1.0	8.6	0.3	10.3
112	34,783	0.0	0.0	0.0	0.7	17.8	0.0	18.6
121	47,154	0.0	0.0	0.0	0.0	3.4	0.0	3.4
122	41,111	0.0	0.0	0.6	0.1	3.1	0.0	3.8
**Totals**	**163,527**	**0.0**	**0.0**	**0.2**	**0.4**	**7.7**	**0.1**	**8.4**
11	40,809	0.0	0.1	0.3	0.0	7.6	0.3	8.3
80	68,774	0.0	0.1	0.2	0.6	4.4	0.6	6.0
								
*Drosophila melanogaster *contigs from major chromosomes								
13	42,664	0.0	0.0	0.0	4.1	3.7	0.0	7.8
112	29,021	0.0	0.2	0.0	0.0	3.7	0.0	3.9
121	46,784	0.0	0.0	0.0	0.0	1.1	0.0	1.1
122	55,801	0.0	0.0	0.0	10.6	1.6	0.0	12.2
**Totals**	**174,270**	**0.0**	**0.0**	**0.0**	**4.4**	**2.3**	**0.0**	**6.8**

Individual contigs from *D. virilis *are shown in each row with the homologous region from *D. melanogaster *shown below. Total size of the contig is shown in bp, followed by percentage of the contig made up of DNA transposons (separated into *1360*, *DINE-1 *and all other DNA transposons), retroelements, simple repeats, and repeats of unknown type (see Materials and methods). Total repeats in the last column is for the contig in that row, and the bold row at the bottom of each group represents the totals for that group. Rows below the bold row represent *D. virilis *contigs where a homologous region from *D. melanogaster *could not be defined, so they were not used in the calculations of total repeat density (see Materials and methods).

**Table 3:** Frequency of individual repetitive elements in *D. virilis*

Repeat element	Class	Number	Average size (bp)	Largest size (bp)	Percent of total repeats
Ten most frequent repeat types (*D. virilis *dot)					
dvir.16.2.centroid	DNA	94	178	1,071	23.4
*Penelope*	LINE	35	199	427	9.8
dvir.2.37.centroid	LTR	1	6,832	6,832	9.6
dvir.0.85.centroid	LINE	8	764	3,711	8.6
dvir.11.33.centroid	Tandem Repeat	31	191	615	8.3
dvir.5.67.centroid	LTR	1	5,406	5,406	7.6
dvir.7.88.centroid	LINE	1	3,370	3,370	4.7
dvir.16.17.centroid	DNA	15	207	739	4.3
dvir.11.23.centroid	Tandem Repeat	13	186	430	3.4
dvir.22.25.centroid	Unknown	6	391	669	3.3
Other					16.9
					
Ten most frequent repeat types (*D. melanogaster *dot)					
DNAREP1_DM (*DINE-1*)	DNA	126	201	631	34.5
yakuba_cons (*DINE-1*)	DNA	54	170	722	12.4
FB4_DM	DNA	8	630	1,215	6.8
PROTOP	DNA	5	938	3,615	6.4
PROTOP_A (*1360*)	DNA	9	518	1,111	6.3
DMCR1A	LINE	8	528	1,253	5.7
PROTOP_B	DNA	14	221	1,083	4.2
TC1_DM (*Tc1*)	DNA	8	343	990	3.7
TART	LINE	5	520	663	3.5
BARI1_DM (*Bari*)	DNA	1	1,728	1,728	2.3
Other					14.0
