# Supplementary figures and images for: Comparison of dot chromosome sequences from D. melanogaster and D. virilis reveals an enrichment of DNA transposon sequences in heterochromatic domains
Source: Genome Biol. 2006 Feb 20;7(2):R15. doi: 10.1186/gb-2006-7-2-r15 (PMC1431729; doi:10.1186/gb-2006-7-2-r15)

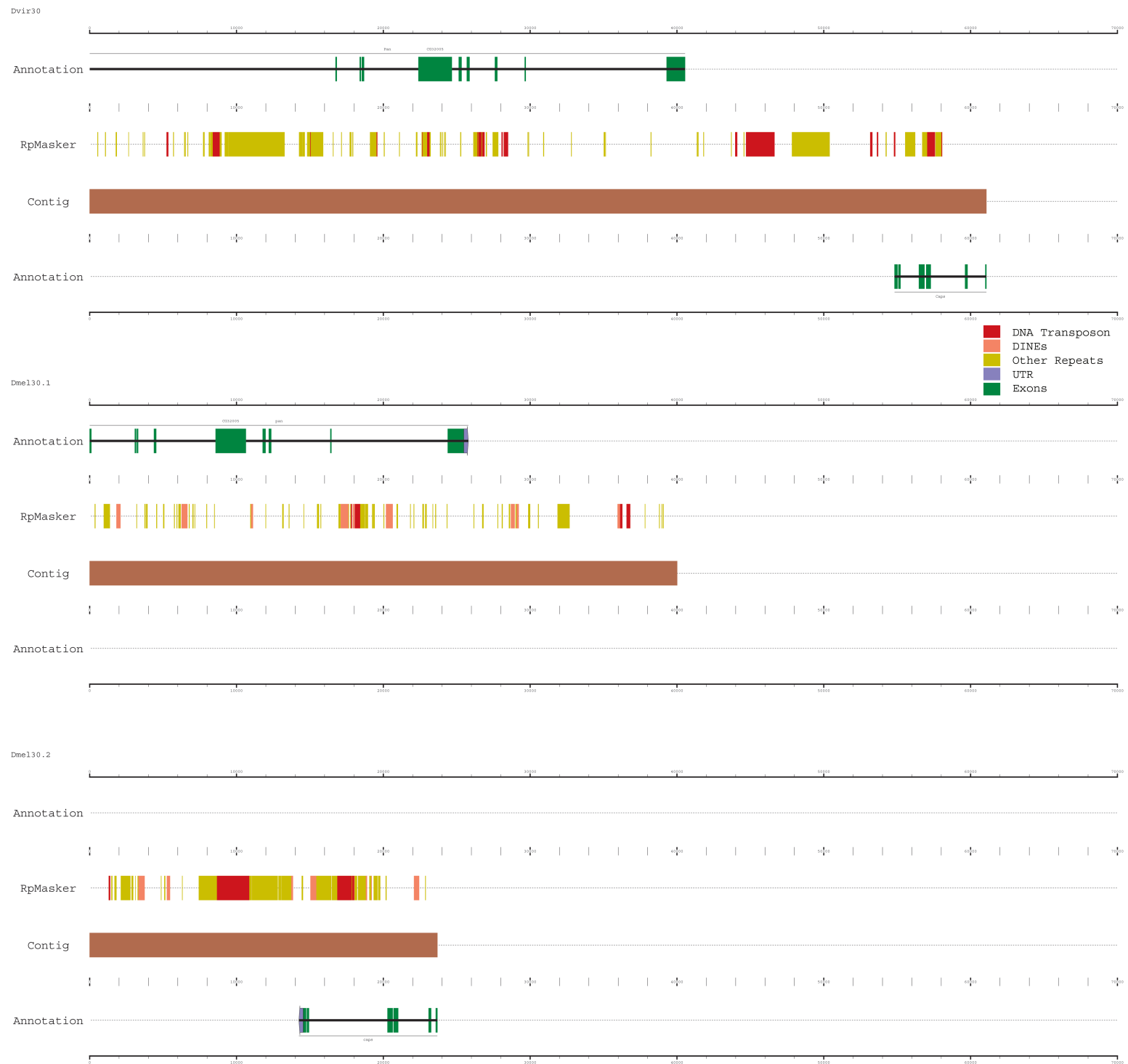

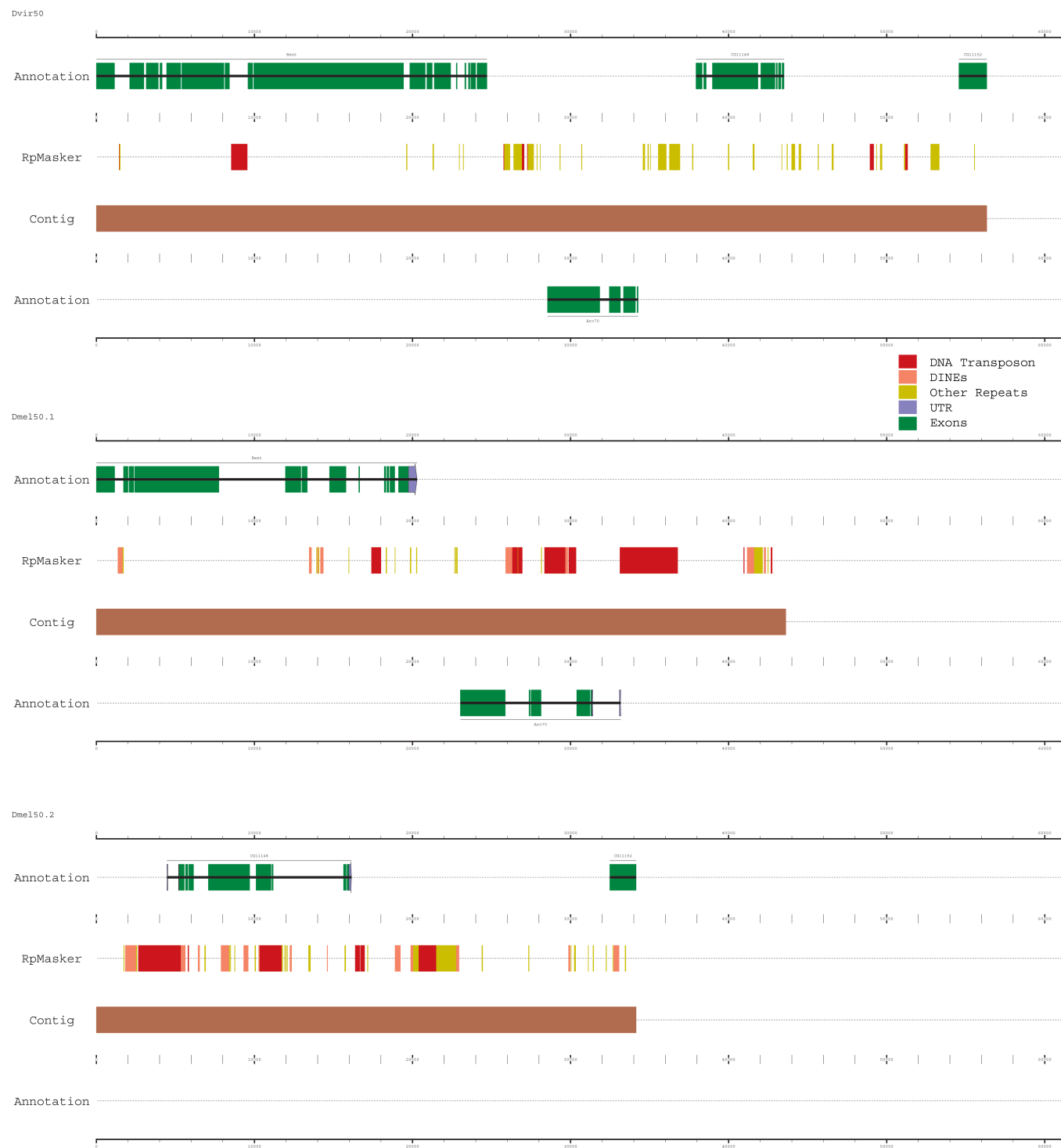

Dvir103

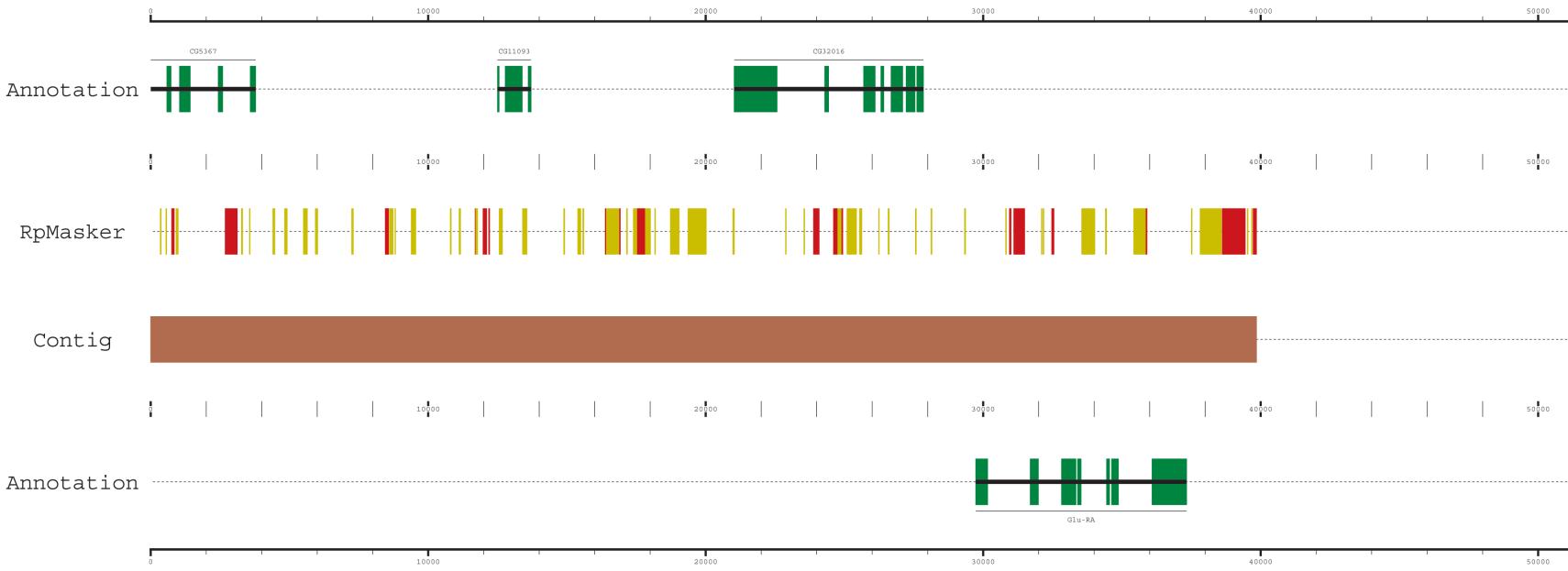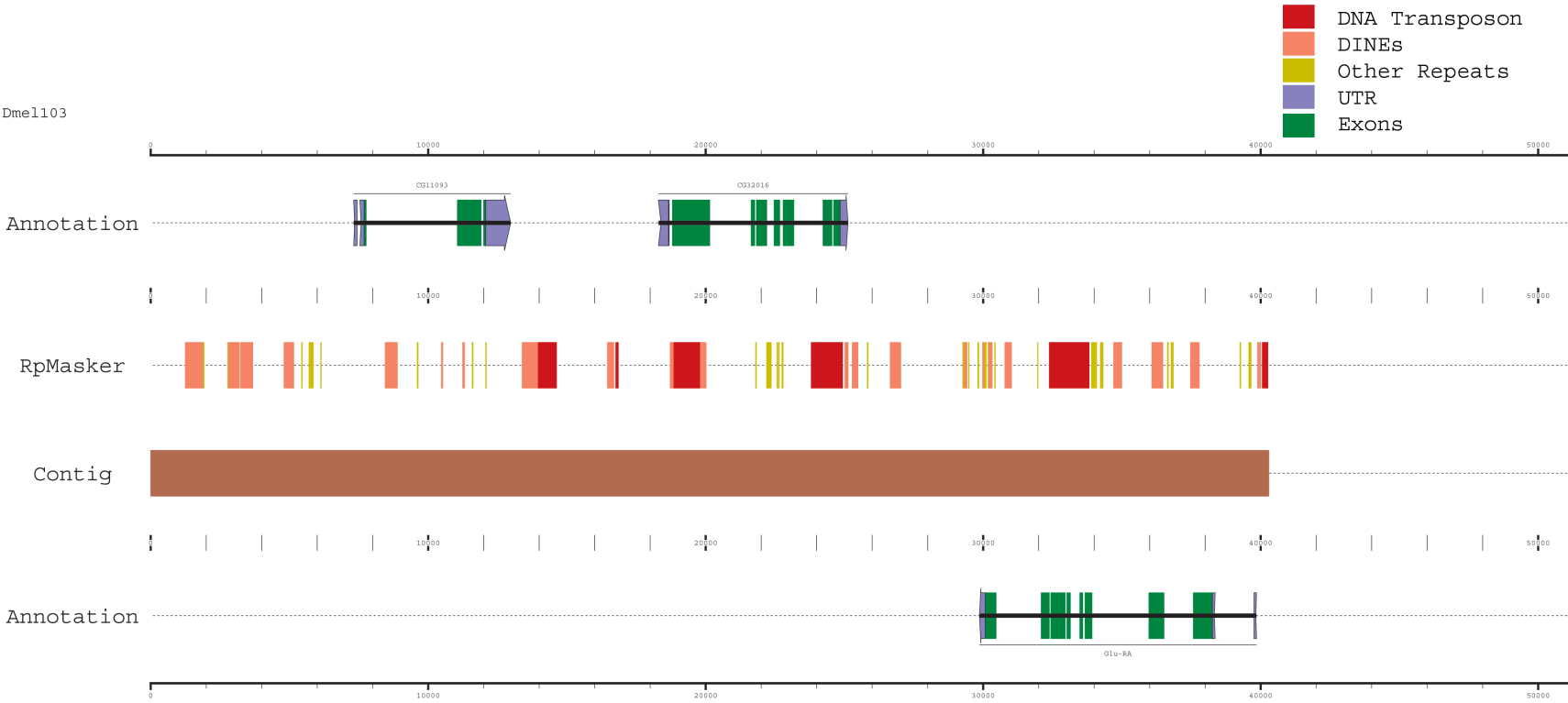

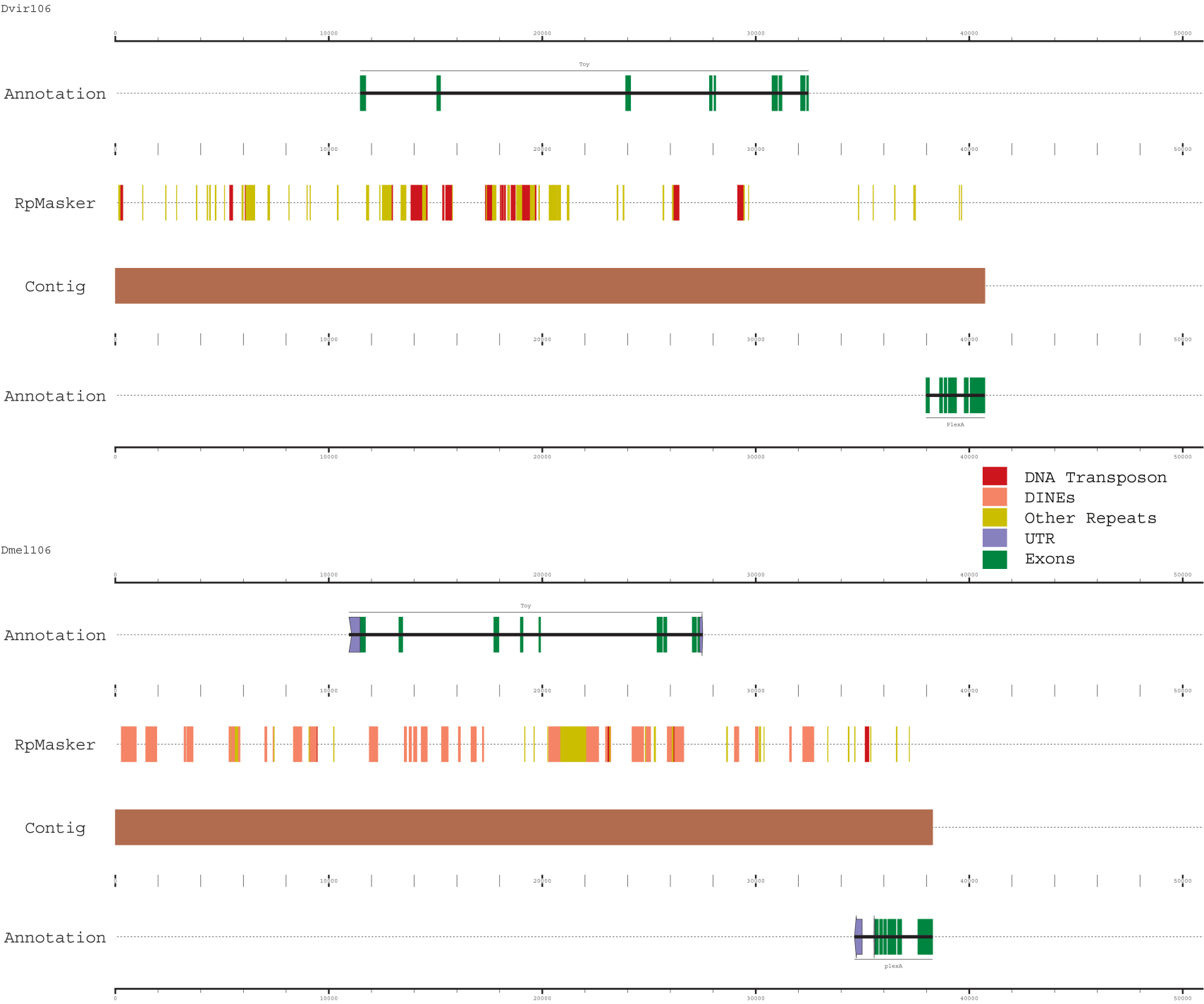

Dvir67

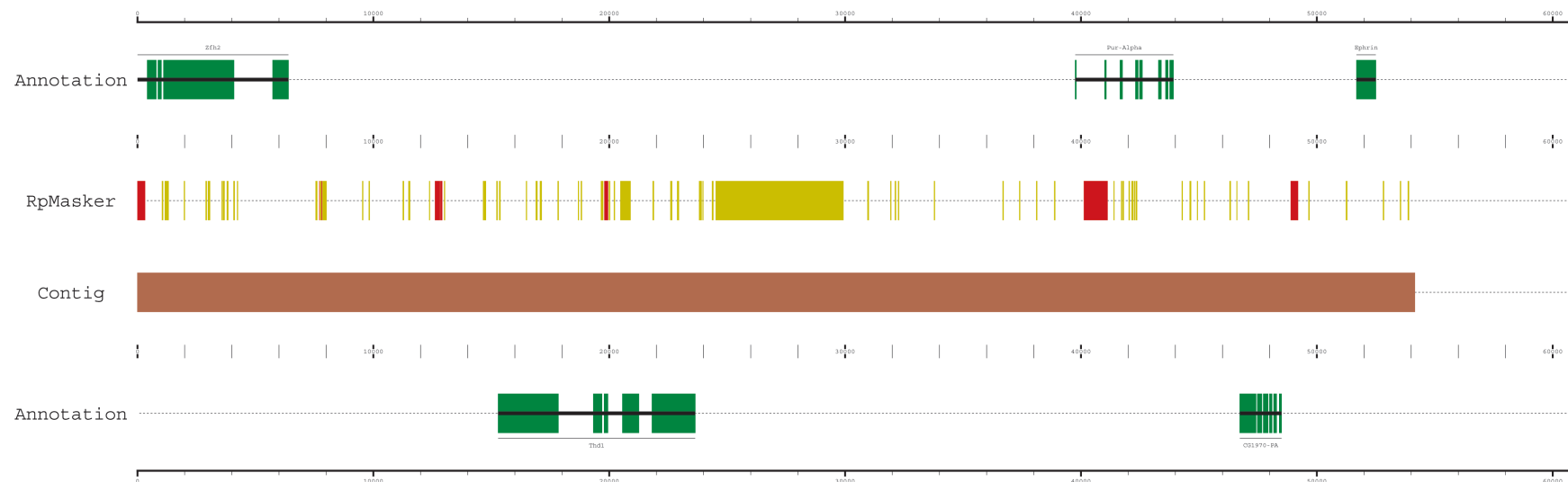

Dmel67

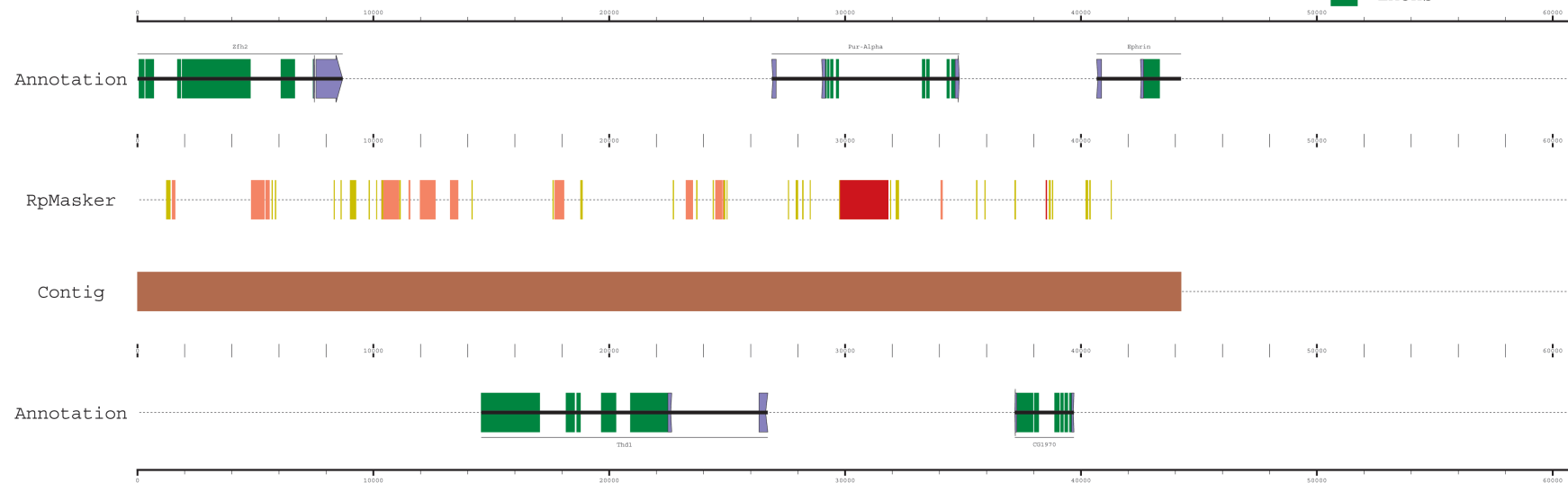

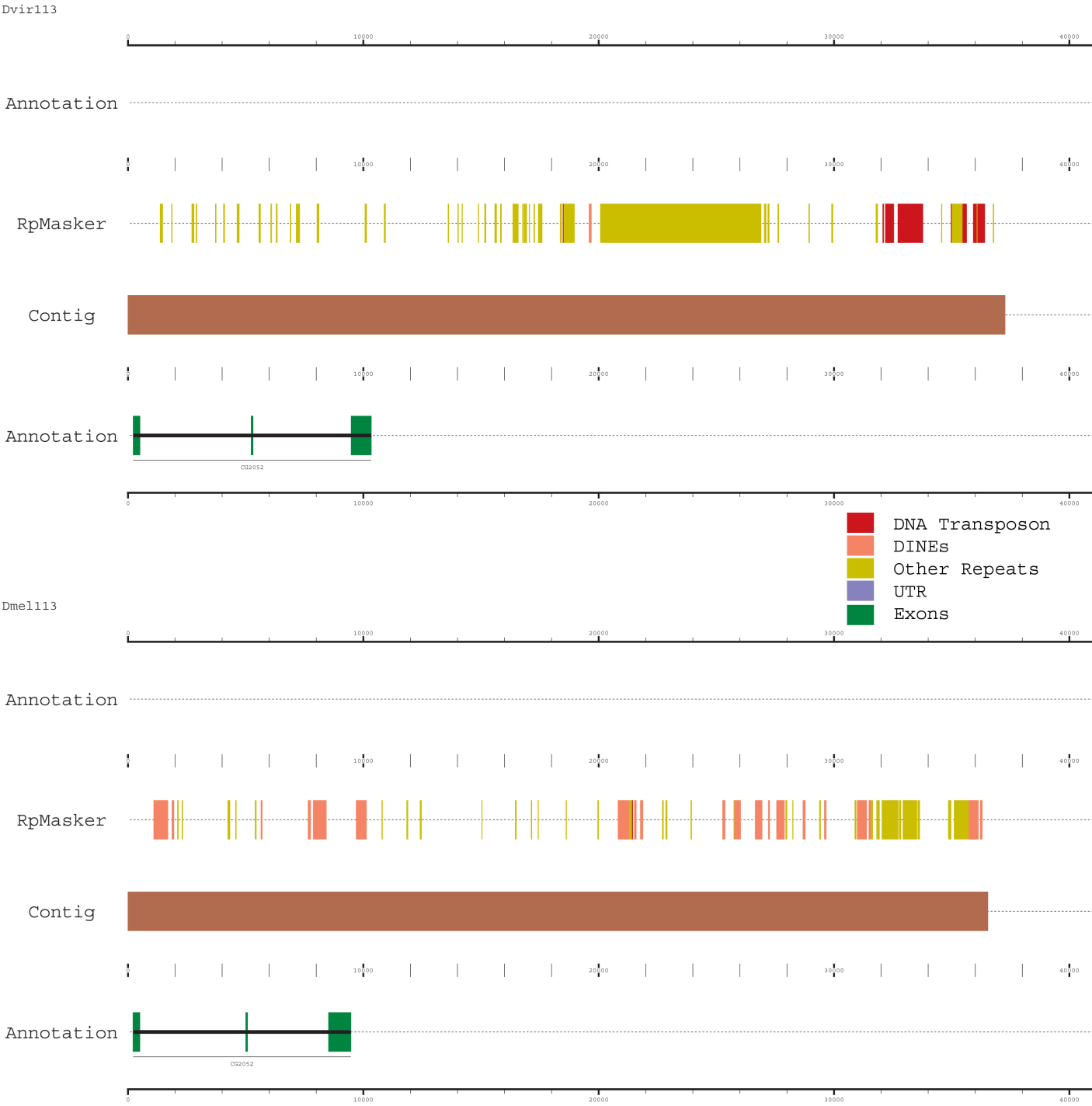

Dvir72

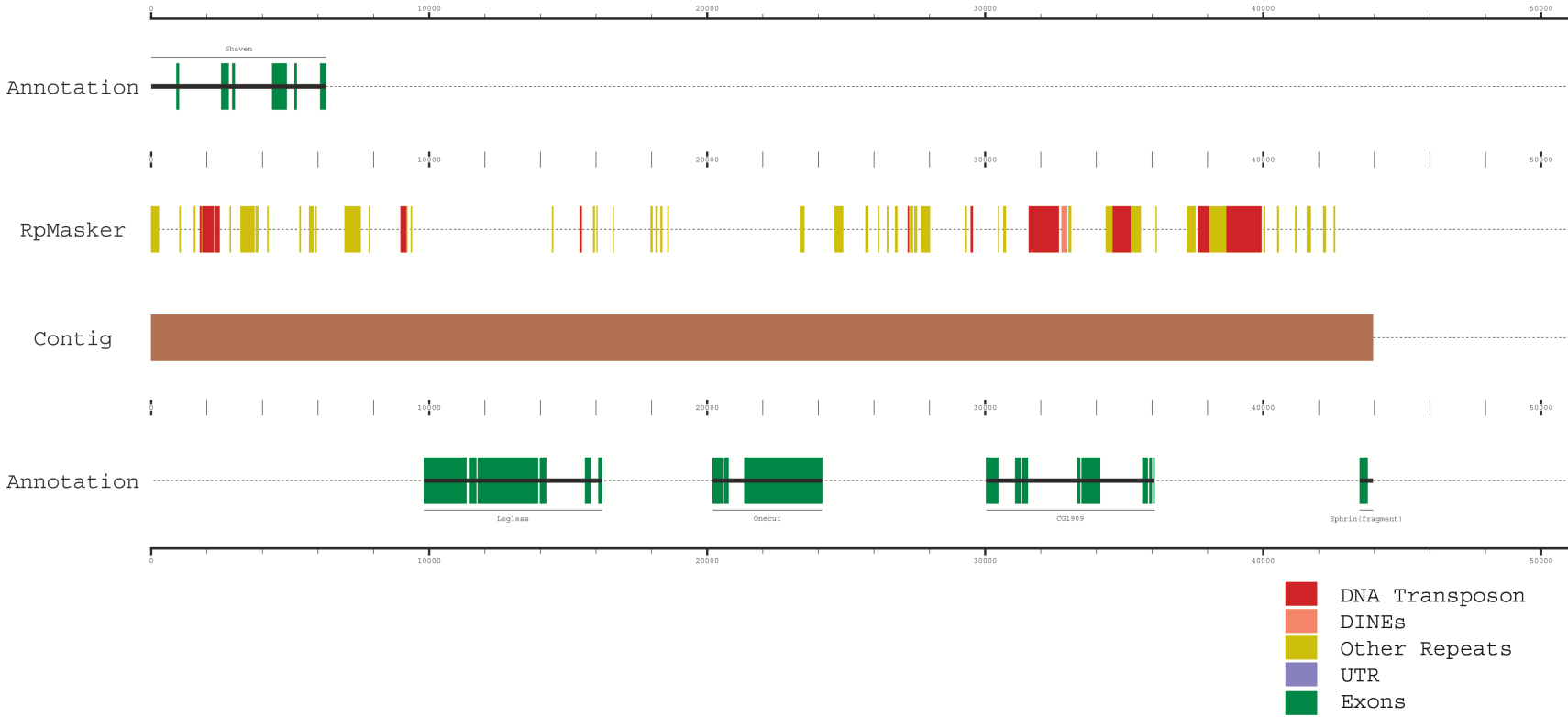

Dvir91

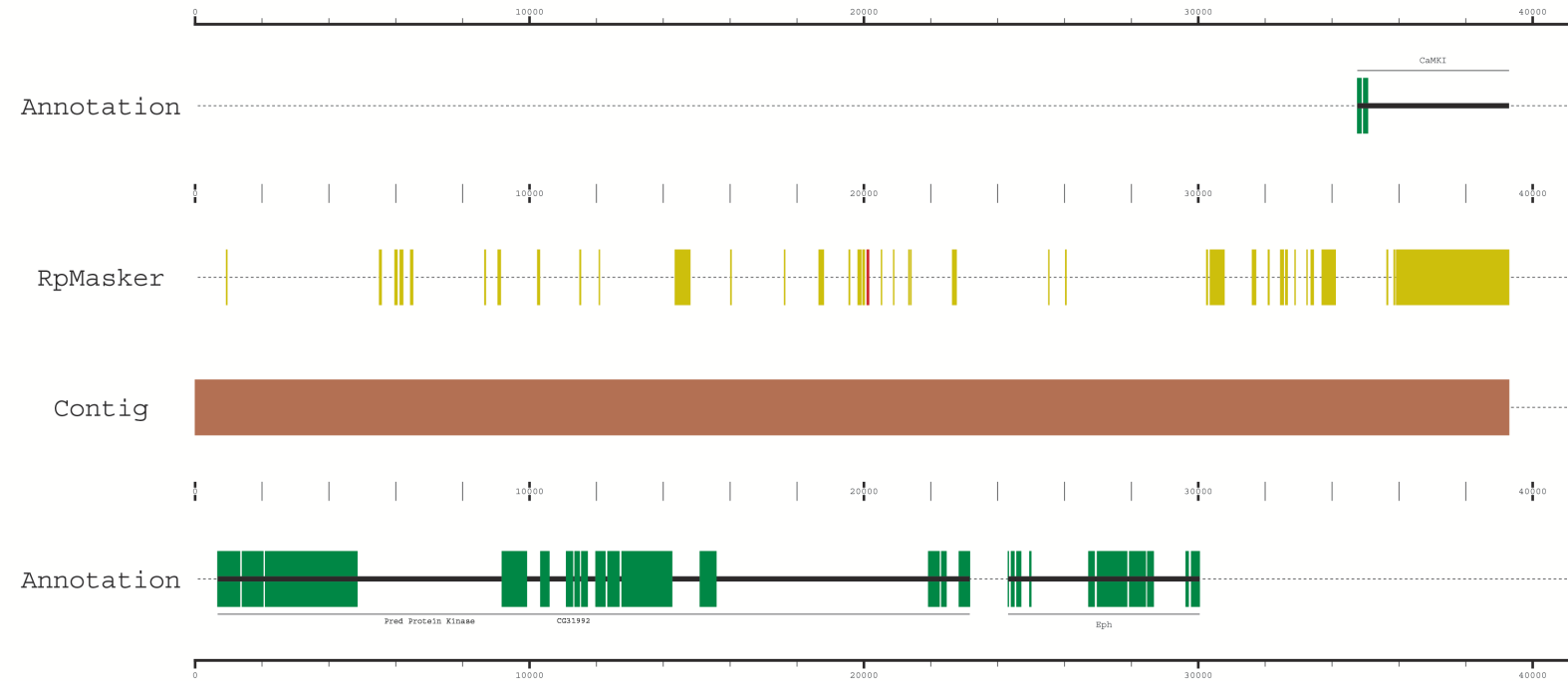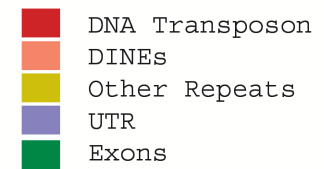

Supplement: Additional data file 1 — Maps of each fosmid from Drosophila virilis and the homologous regions from Drosophila melanogaster (if available) showing the genes and identified repetitive elements for dot chromosome sequences [file gb-2006-7-2-r15-S1.pdf]
