# Supplementary material for: Comparison of dot chromosome sequences from D. melanogaster and D. virilis reveals an enrichment of DNA transposon sequences in heterochromatic domains
Source: Genome Biol. 2006 Feb 20;7(2):R15. doi: 10.1186/gb-2006-7-2-r15 (PMC1431729; doi:10.1186/gb-2006-7-2-r15)
Supplement: Additional data file 2 — Maps of each fosmid from D. virilis and the homologous regions from D. melanogaster (if available) showing the genes and identified repetitive elements for non-dot chromosome sequences [file gb-2006-7-2-r15-S2.pdf]

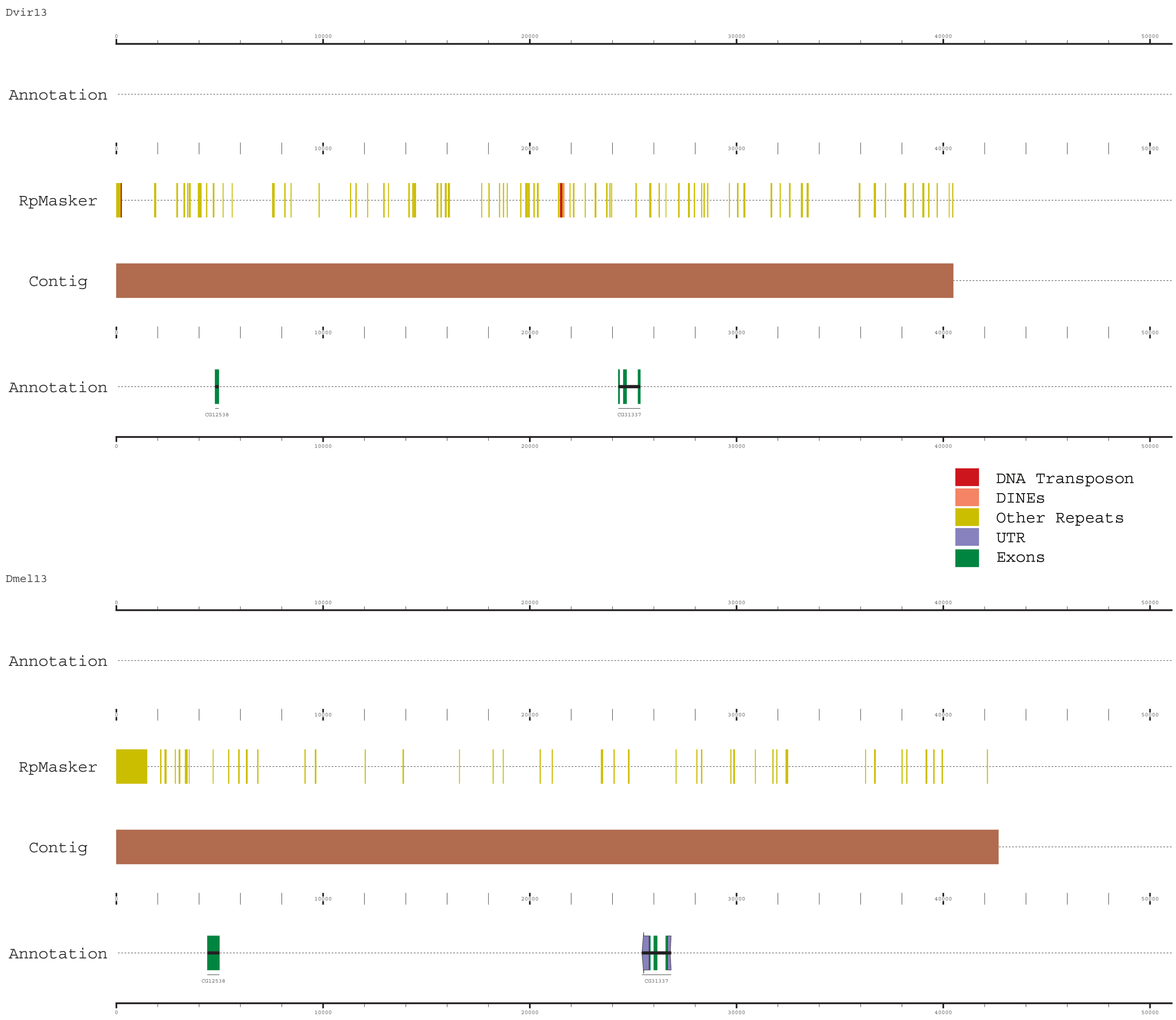
Dvir13
0
10000
20000
30000
40000
50000
Annotation
RpMasker
Contig
Annotation
CG12538
CG31337
DNA Transposon
DINEs
Other Repeats
UTR
Exons
Dmel13
Annotation
RpMasker
Contig
Annotation
CG12538
CG31337

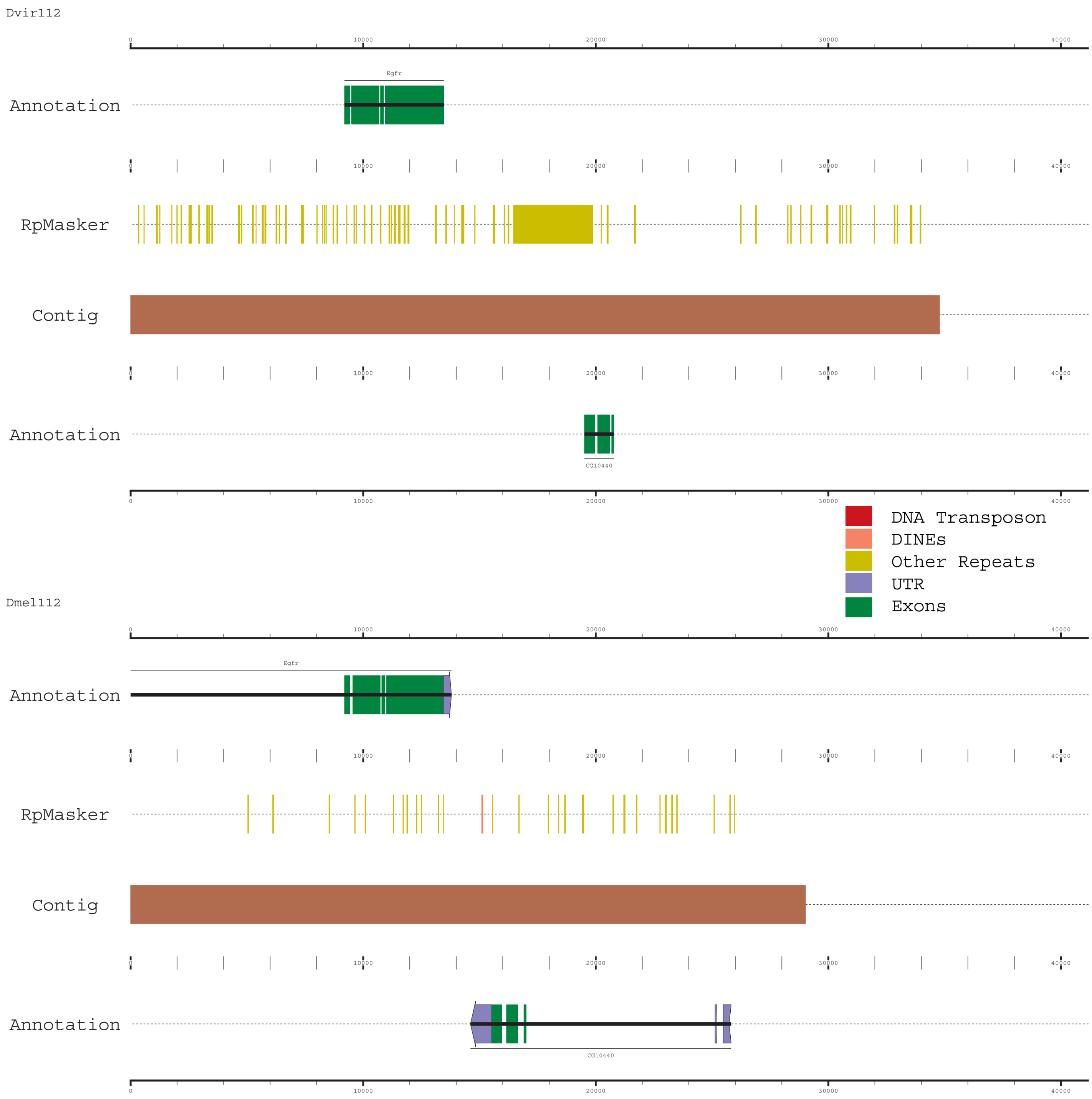

Dvir112
0
10000
20000
30000
40000
Egfr
Annotation
RpMasker
Contig
Annotation
CG10440
DNA Transposon
DINEs
Other Repeats
UTR
Exons
Dmel112
Egfr
Annotation
RpMasker
Contig
Annotation
CG10440

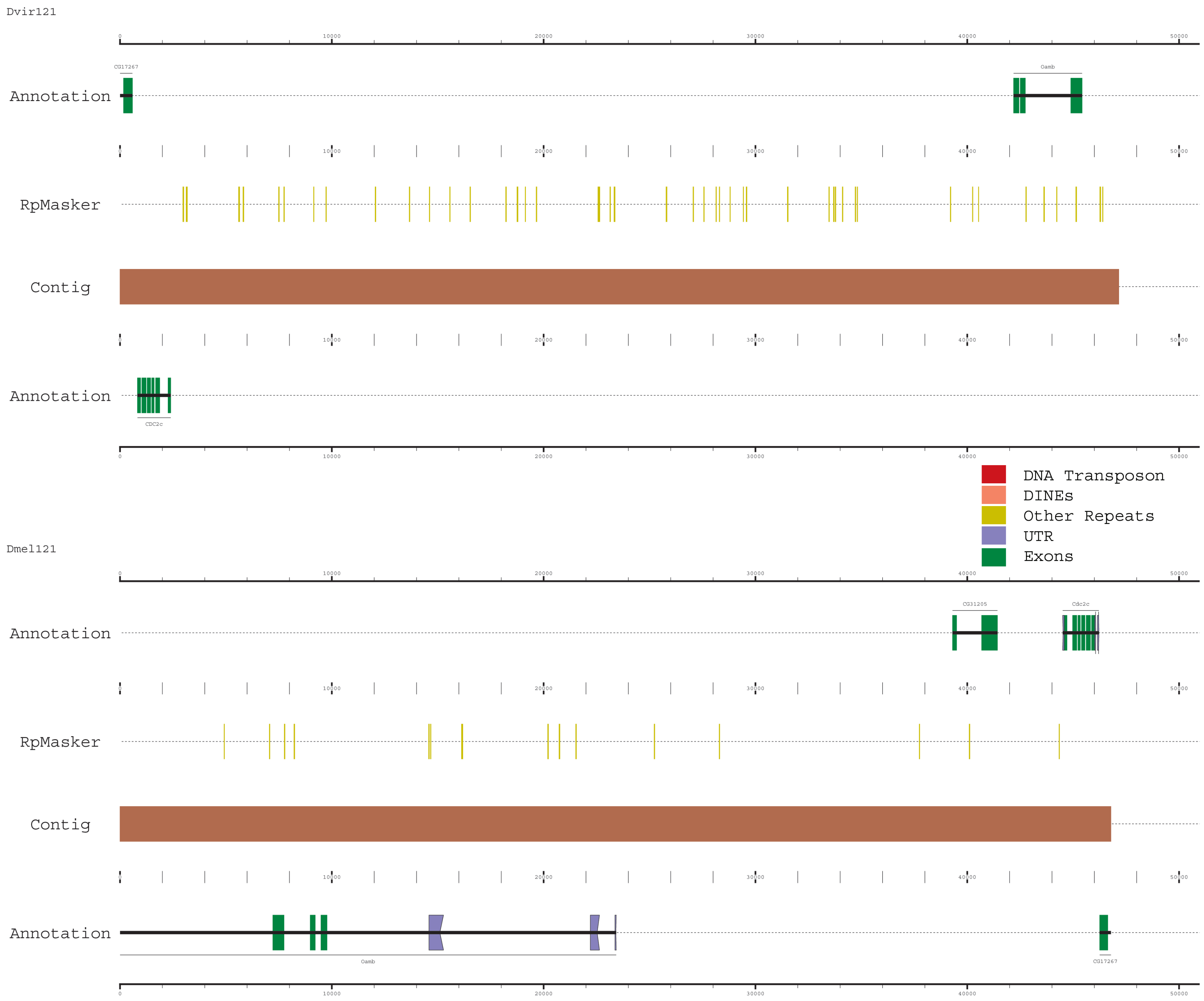

Dvir121
0
10000
20000
30000
40000
50000
Annotation
CG17267
Oamb
RpMasker
Contig
Annotation
CDC2c
DNA Transposon
DINEs
Other Repeats
UTR
Exons
Dmel121
Annotation
CG31205
Cdc2c
RpMasker
Contig
Annotation
Oamb
CG17267

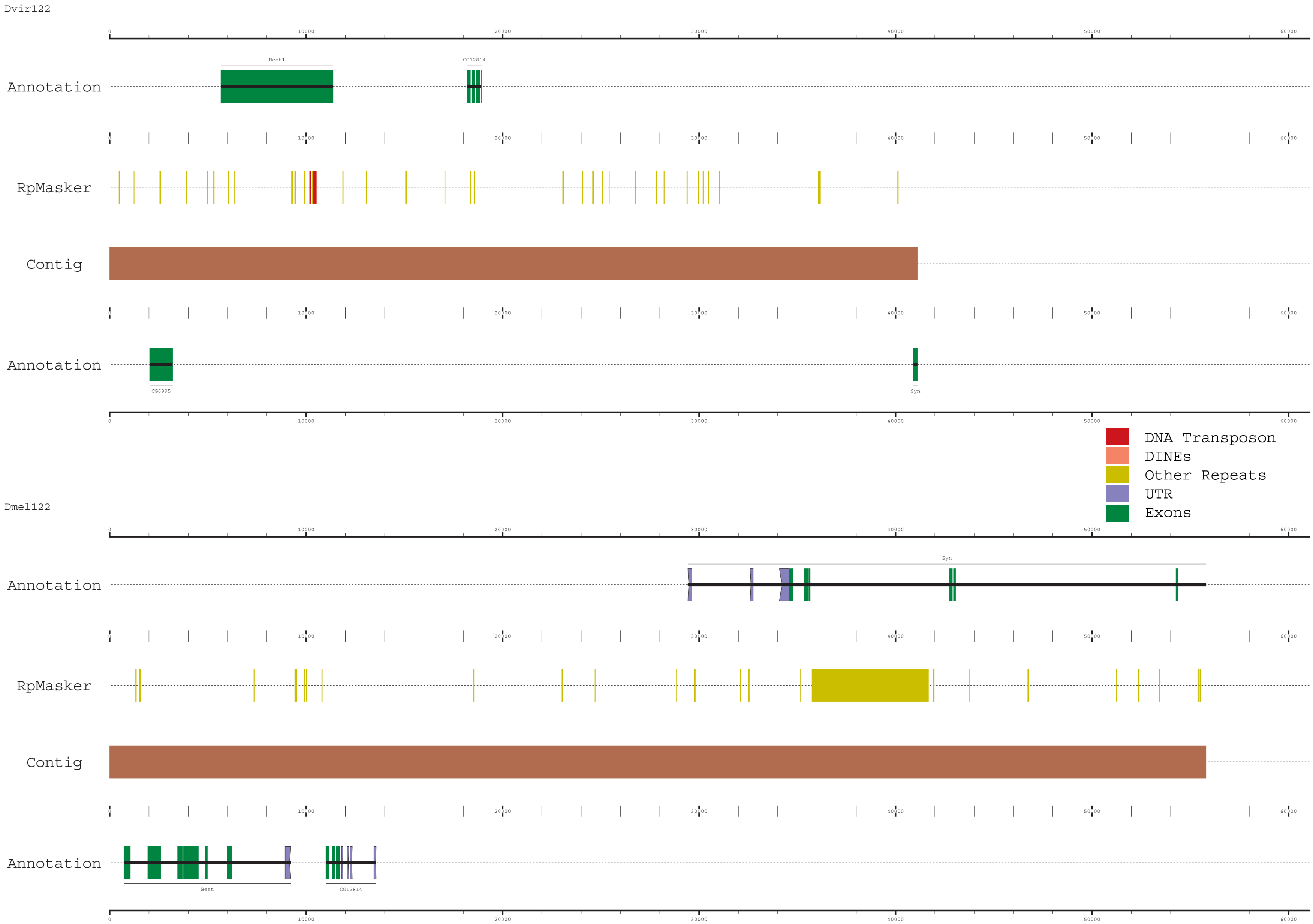
Dvir122
Annotation
Best1
CG12814
RpMasker
Contig
Annotation
CG6995
Syn
DNA Transposon
DINEs
Other Repeats
UTR
Exons
Dmel122
Annotation
Syn
RpMasker
Contig
Annotation
Best
CG12814
0
10000
20000
30000
40000
50000
60000

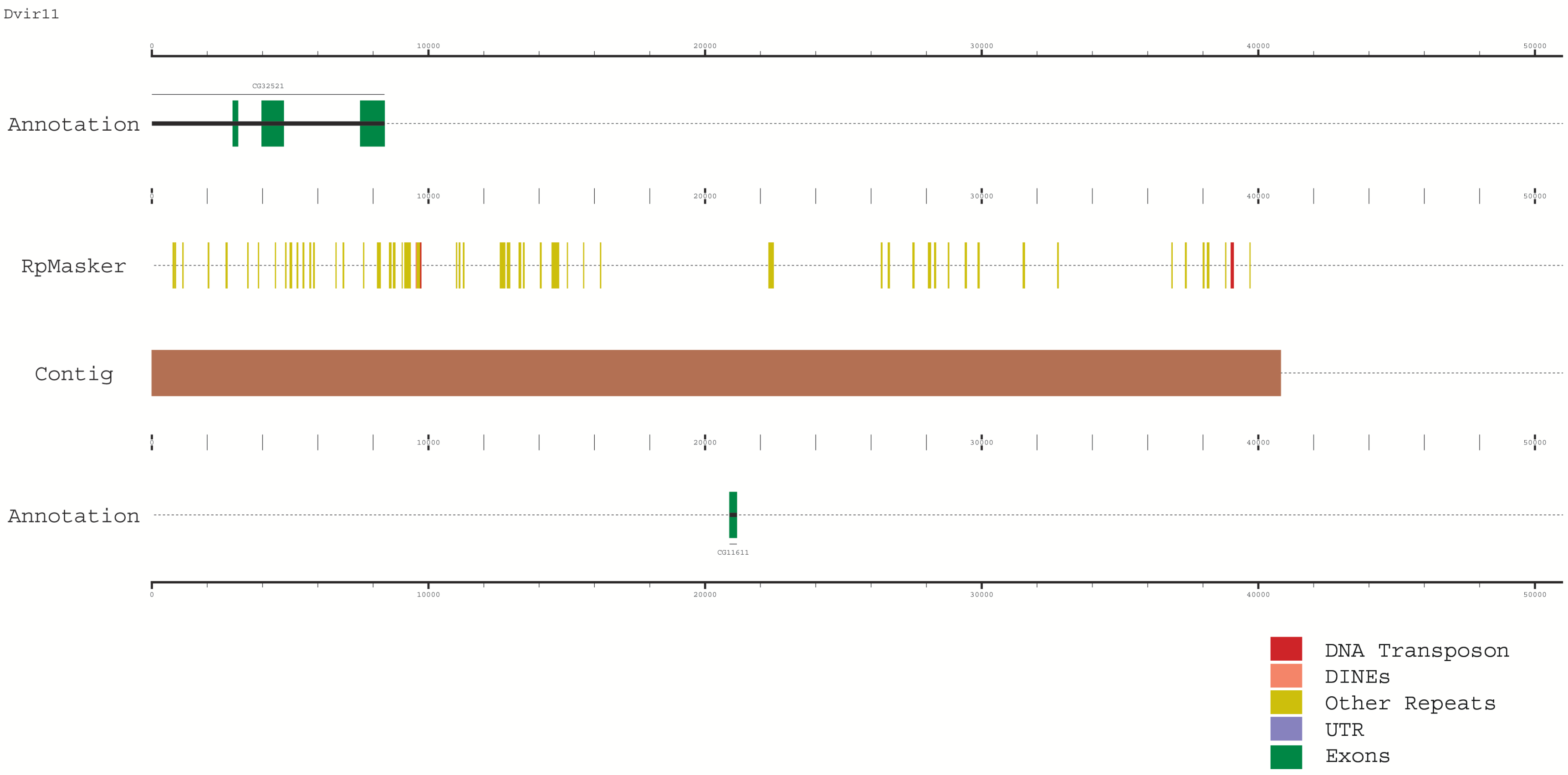
Dvir11
0
10000
20000
30000
40000
50000
CG32521
Annotation
RpMasker
Contig
Annotation
CG11611
DNA Transposon
DINEs
Other Repeats
UTR
Exons

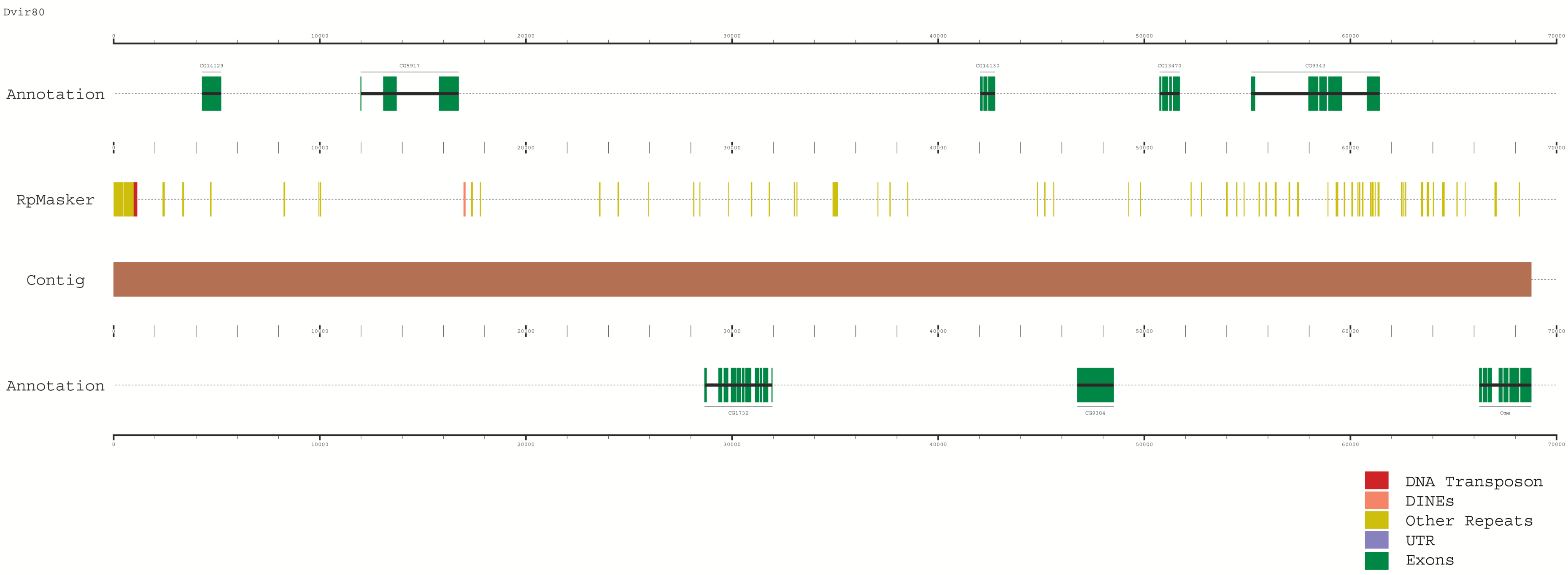
Dvir80
Annotation
RpMasker
Contig
Annotation
CG14129
CG5917
CG14130
CG13470
CG9343
CG1732
CG9384
Ome
0
10000
20000
30000
40000
50000
60000
70000
DNA Transposon
DINEs
Other Repeats
UTR
Exons
